# Promoting New Approach Methodologies (NAMs) for research on skin color changes in response to environmental stress factors: tobacco and air pollution

**DOI:** 10.3389/ftox.2023.1256399

**Published:** 2023-10-10

**Authors:** Katherine Virginia Bouchard, Gertrude-Emilia Costin

**Affiliations:** Institute for In Vitro Sciences, Inc. (IIVS), Gaithersburg, MD, United States

**Keywords:** air pollution, tobacco smoke, skin pigmentation, New Approach Methodologies, skin aging

## Abstract

Aging is one of the most dynamic biological processes in the human body and is known to carry significant impacts on individuals’ self-esteem. Skin pigmentation is a highly heritable trait made possible by complex, strictly controlled cellular and molecular mechanisms. Genetic, environmental and endocrine factors contribute to the modulation of melanin’s amount, type and distribution in the skin layers. One of the hallmarks of extrinsic skin aging induced by environmental stress factors is the alteration of the constitutive pigmentation pattern clinically defined as senile lentigines and/or melasma or other pigmentary dyschromias. The complexity of pollutants and tobacco smoke as environmental stress factors warrants a thorough understanding of the mechanisms by which they impact skin pigmentation through repeated and long-term exposure. Pre-clinical and clinical studies demonstrated that pollutants are known to induce reactive oxygen species (ROS) or inflammatory events that lead directly or indirectly to skin hyperpigmentation. Another mechanistic direction is provided by Aryl hydrocarbon Receptors (AhR) which were shown to mediate processes leading to skin hyperpigmentation in response to pollutants by regulation of melanogenic enzymes and transcription factors involved in melanin biosynthesis pathway. In this context, we will discuss a diverse range of New Approach Methodologies (NAMs) capable to provide mechanistic insights of the cellular and molecular pathways involved in the action of environmental stress factors on skin pigmentation and to support the design of raw ingredients and formulations intended to counter their impact and of any subsequently needed clinical studies.

## 1 Introduction

Aging is a complex of progressive, irreversible and unavoidable structural and functional changes that the human body naturally progresses through, all highly dependable on and influenced by intrinsic and extrinsic factors within or outside our control. Deciphering the complicated pathways responsible for aging and discovering possibilities to delay the process has been a challenging task for many researchers. Skin aging in particular is of interest and concern to the medical field since it has multiple immediate medical, psychological and social impacts on one’s self-esteem as skin’s unique texture and color may change significantly in response to stress factors (Gupta and Gilchrest, 2005).

In this review, we will focus on the activity and impact of extrinsic factors that contribute to skin aging, specifically to skin pigmentation. Of these factors, growing evidence demonstrate that environmental aggressors contribute to skin aging through induction of hyperpigmentation among several other pathways. Based on the available studies, we will primarily address the effects induced to skin pigmentation by prolonged or repetitive exposure to tobacco, Particulate Matter (PM) [including Diesel Exhaust Particles (DEPs)], Polycyclic Aromatic Hydrocarbons (PAHs), and to a lesser extent Polychlorinated Biphenyls (PCBs).

Through our review of available sources, we identified 16 manuscripts reporting on the action of air pollution on skin pigmentation, and 11 on tobacco’s impact. The search and identification of these manuscripts was conducted using primarily the PubMed database and the individual websites of the journals the papers were published in. The search terms used to narrow the resources as pertaining to the topic of the manuscript were: “*in vitro*”, “NAMs”, “*in vivo*”, “clinical”, “*ex vivo*”, “melanocytes”, “keratinocytes”, “3D model”, “melanogenesis”, “melanin synthesis”, “skin pigmentation”, skin aging”, “pollution”, “smoking”, “tobacco”, “tobacco smoke”, “nicotine”, “smoking”. As for the inclusion criteria, the manuscripts considered for our analysis had to associate the pollutants (of choice, see exclusion criteria) and tobacco (and associated terms) to their mechanism of action being investigate and/or to products being researched for their anti-pollution and anti-tobacco-induced aging skin potential. Also as an inclusion criteria, only the manuscripts investigating specifically skin pigmentation as sign of skin aging were considered. The manuscripts that met the following exclusion criteria were not considered for our analysis: non peer-reviewed; investigating pollutants other than PM, PAHs, PCBs, DEPs; introducing a hypothetical mechanism not supported at least by preliminary data or by cross-referenced studies (entirely speculative); investigated the impact of external factors on skin pigmentation other than pollutants and tobacco (as narrowed down by these criterion) given the scope of the manuscript. Our analysis identified a tendency to use clinical studies, especially to research tobacco’s impact on skin pigmentation. Clinical studies are designed to investigate the effects of various toxic agents on skin at a visual, measurable level, thus providing valuable epidemiological evidence. They are however limited in their capacity to shed light on the cellular and molecular mechanisms which are key to the design of products to counteract the deleterious effects of these toxicants. Besides being relatively complex and expensive to conduct, clinical studies are more difficult to standardize in terms of design to fit the goals and endpoints of interest. Furthermore, clinical studies researching skin pigmentation are even more challenging to set up as they need to eliminate or limit the contribution of confounding factors (*e.g*., UV) to melanin production.

Our analysis also identified an encouraging trend in the reduction of animal-based studies focused on the impact of air pollution on skin pigmentation. In general, rodents (*i.e.*, rats or mice) or pigs represent the main *in vivo* test systems used to study skin responses to toxicants (Abd et al., 2016). Rodent skin differs significantly from human skin in terms of thickness and higher density of hair follicles which subsequently increase dermal penetration (Todo, 2017). In comparison, the porcine skin resembles structurally the properties of human skin (Debeer et al., 2013), however the animals are more challenging to handle and have higher fat storage (Summerfield et al., 2015), which could be a confounding factor as many pollutants are stored in the hypodermis (La Merrill et al., 2013). Finally, research on skin aging is relatively difficult to replicate in an animal test system which makes the observations very challenging, if not impossible. Therefore, aging is often induced chemically or biologically in an animal-based research setting. As a consequence, this approach is less reliable and more difficult to execute compared to the convenience of smaller scale but quite complex platforms based on alternative test systems.

Advances in the toxicological methodologies and increasing ethical concerns regarding animal experimentation led to the development of various alternative methods based on the Replacement, Reduction and Refinement (3Rs) of animal studies (Naik et al., 2023). In our review we identified that more than 50% of the reported experiments were conducted using non-animal test systems to investigate the impact of environmental stress factors on skin pigmentation. This is a very encouraging trend which should be further supported and promoted to help expand the research on tobacco’s impact on skin color. The design of these experiments facilitates an in-depth understanding of the mechanisms involved in the impact of air pollution and tobacco smoke on skin pigmentation and aging process. It is our opinion that this level of experimental complexity can be accomplished by using modern New Approach Methodologies (NAMs) which are based on *in chemico*, *in vitro*, and *ex vivo* test system models. These technologies offer endless possibilities to gain a detailed understanding of the mechanisms of action and also to design potent and safe actives capable to counteract the damaging action of aggressors and to inform future directions of clinical studies, as detailed in this review. We will also provide insights into study design, selection of appropriate test system, type of toxicant, route and length of exposure, and human exposure relevance. Last but not least, we will discuss possible future research directions into untapped mechanisms which may hold the key to efficient treatments addressing skin hyperpigmentation induced by pollutants.

## 2 Extrinsic skin aging induced by environmental stress factors: impact on skin pigmentation

The skin is a complex organ responsible for multiple functions that are critical for the overall body physiology such as protection (against biological, mechanical or chemical factors), sensation (touch, temperature, pain, pressure), regulation of temperature, absorption and excretion, synthesis of vitamin D and aesthetic and social functions. Extrinsic and intrinsic factors act in concert upon the physiology and functionality of the largest organ in the human body and are responsible for skin aging. Extrinsic skin aging is induced by stressors originating in the environment and is typified by coarse wrinkles, elastosis and irregular pigmentation.

Besides the obvious changes aging brings in one’s life quality, this global phenomenon is highly dependent on the sense of perception and self-awareness that seems to have increased in recent decades, alongside individuals’ desire to improve their appearance by maintaining a healthy skin and delaying the aging process. Signs of skin aging, such as senile lentigines (age spots), melasma or other pigmentary dyschromias motivate aging population to seek treatment for these conditions. While for a long time exposure to sun was considered the main contributory factor to aging, with direct impact on skin pigmentation, recent studies point towards other detrimental external factors such as air pollution. The World Health Organization (WHO) reports that air pollution is the most significant and urgent public problem of the 21^st^ century (WHO, 2021). Although tobacco smoke is technically considered part of the air pollution, for the purpose of this review we will treat them separately as they relate to distinct human behaviors. While exposure to tobacco smoke can be considered a voluntary behavior and therefore controllable (with the exception of secondhand smoke exposure), exposure to air pollution is often involuntary and less or not at all controlled.

### 2.1 Air pollution

High pollution levels were initially identified in urban areas, however they became a global concern and the world’s largest single environmental health risk factor associated with millions of deaths annually (WHO, 2021). The anti-pollution movement in cosmetic and personal care industry began in the Asia Pacific region and it has now reached the western markets, launching hundreds of commercially successful products addressing this global concern (Pitman, 2017). One of the targets for these products is skin hyperpigmentation as the production of melanin is a very sensitive marker to changes induced by stress factors, including pollution. Integration of the complex biological pathway of melanin biosynthesis in the world wide context of skin tone uniqueness combined with geographically specific pollution concerns makes the research of the molecular mechanisms involved even more important.

The source and composition of pollutants are important aspects to take into account when conducting research focused on their effects on skin pigmentation. Air pollutants are emitted directly from the source (volcanic eruptions, forest fires, biological decay, power plants, industries, car emissions, fossil fuel burning, etc.) or following exposure to heat or UV (Krutmann et al., 2014). PM is a key component of air pollution that has been thoroughly investigated. The particle size, concentration and chemical properties of PM vary widely geographically and in time (Kampa and Castanas, 2008). Because of their textured structure, the particles have an ideal surface for the attachment of other toxicants such as PAHs, which originate from wood burning, combustion of organic material including coal burning, automobile exhaust fumes, and cigarette smoke. PAHs are highly lipophilic and therefore can easily penetrate through the skin barrier (Jux et al., 2011) and reach the melanocytes, the pigment producing cells resident in the skin. Multiple studies have shown that exposure to other air pollutants such as DEPs is associated with clinical signs of hyperpigmentation (Krutmann et al., 2020; Grether-Beck et al., 2021). Last but not least, PCBs are highly persistent environmental pollutants known to have accidentally contaminated rice oil alongside polychlorinated dibenzofurans (PCDFs) and polychlorinated quaterphenyls (PCQs) (Yusho accident, Japan, 1968 and Yu-Cheng accident, Taiwan, 1979) (Masuda, 1985). One of the symptoms for the poisoning that occurred during these incidents, though not the most impactful in terms of toxic effects, was increased skin pigmentation.

Impact of air pollution on animal species has been the subject of observations in populated areas and provided more evidence into the long-term effects on skin. For example, increased levels of pigmentation were observed to be common in several animal species (*e.g.*, pigeons and moths) found in urban industrial environments (Chatelain et al., 2014; Van’t Hof et al., 2016). Observations on *Emydocephalus annulatus* showed that overproduction of melanin occurred more frequently in skin of sea snakes living in polluted water compared to unpolluted environments (Goiran et al., 2017). These observations support the contribution of pollution to skin hyperpigmentation.

The ability of particles to penetrate the skin layers is still debatable especially since studies report conflicting results likely due to having used a variety of particle types and different experimental models and designs (Toll et al., 2004; Baroli et al., 2007). Ambient PM was shown to penetrate the skin either following the route of hair follicles and sweat ducts (Lademann et al., 2004; Koohgoli et al., 2017) or transepidermally (Jin et al., 2018). Thus, they may be able to reach the melanocytes and release the highly lipophilic and toxic surface-bound PAHs. Air pollutants are also capable to affect directly the function of skin cells by hijacking classic signaling pathways otherwise known to be UV’s routes of action on skin pigmentation. It was recently demonstrated that particles can also penetrate the *stratum corneum* (Burke, 2018). Further mechanistic studies are necessary to elucidate the contribution of the particles and other attached environmental stress factors to extrinsic skin aging in order to find reliable means to counteract their actions.

### 2.2 Tobacco smoke

Of an equally complex composition to air pollutants is the tobacco smoke, an aerosolized by-product of tobacco combustion during the smoking of cigarettes and related products and consisting of a particulate and a gas phase. While nicotine is known to be absorbed through skin (Benowitz and Hukkanen, 2009), details regarding the penetration of other fractions of the tobacco smoke through dermal layers is lacking. Even though nicotine is easy to use on its own in research experiments, the reality indicates that the most harmful toxicants are found primarily in the gas phase. It is therefore of the utmost importance to perform hazard and risk assessment using a combination of both phases to better represent the in-use exposure and to take into consideration any synergistic effects based on the interactions between the two phases. The particulate phase may include PAHs and tobacco specific byproducts, while the gas phase may contain dioxins among other harmful radicals. Of the dioxins, 2,3,7,8-tetrachlorodibenzo para-dioxin (TCDD) was demonstrated to be the most toxic molecule and one of the first environmental-related molecule shown to stimulate skin pigmentation (Luecke et al., 2010), thus opening up the investigations into premature skin aging induced by tobacco (Kennedy et al., 2003; Morita et al., 2009).

### 2.3 Melanin biosynthesis pathways

Melanocytes are highly specialized cells responsible for melanin biosynthesis through a complex pathway hosted by membrane-bound organelles known as melanosomes (Hearing, 1997). Following four stages of maturation, the melanosomes are transferred through the dendrites of the melanocytes to the surrounding keratinocytes where they play a critical role in photo-protection. The established “epidermal melanin unit” is comprised of a ratio of one melanocyte to 40 keratinocytes in the basal and suprabasal layers (Ostrowski and Fisher, 2019). The epidermal melanin unit responds rapidly to a multitude of environmental stimuli, of which UV was identified early as one of the main contributing factors to stimulate the melanin production. Melanocytes respond directly to UV exposure and through their close communication with the keratinocytes.

**FIGURE 1:**
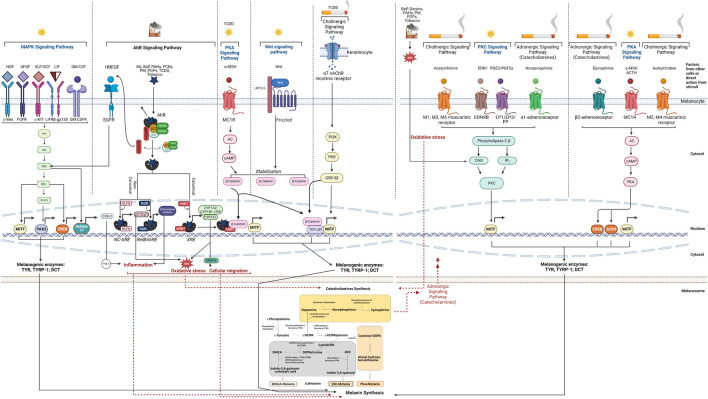
Overview of demonstrated or possible mechanisms and pathways involved in melanin synthesis induced by air pollution and tobacco. The mechanisms are depicted to take place within the melanocytes and in their specific cellular compartments (cytosol, nucleus and melanosome), except where the participation of other cells (*i.e.*, keratinocytes) is indicated. Abbreviations: AC, Adenylate Cyclase; ACTH, Adenochorticoprotic Hormone; AhR, Aryl hydrocarbon Receptor; ARNT, Aryl Hydrocarbon Receptor Nuclear Translocator; BA, Benzanthrone; BaP, benzo[a]pyrene; bFGF, basic Fibroblast Growth Factor; cAMP; cyclic Adenosine Monophosphate; CREB, cAMP Response Element-Binding Protein; COX, Cyclooxygenase; CYP, cytochrome; DAG, Diacylglycerol; DCT, DOPAchrome Tautomerase; DHI, 5,6-dihydroxyindole; DHICA, 5,6-dihydroxyindole-2-carboxylic acid; DOPA, L-3,4-dihydroxyphenylalanine; EDN1 (EDN-1, ET-1), Endothelin-1; EGF, Epidermal Growth Factor; EDNRB, Endothelin Receptor Type B; EGFR, Epidermal Growth Factor Receptor; EP, Prostaglandin E Receptor; ERK, Extracellular-signal Regulated Kinase; FGFR, Fibroblast Growth Factor Receptor; FP, Prostaglandin F Receptor; GM-CSF, Granulocyte-Macrophage Colony-Stimulating Factor; GM-CSFR, Granulocyte-Macrophage Colony-Stimulating Factor Receptor; GSK-3β, Glycogen Synthase Kinase 3 beta; HBEGF, Heparin Binding EGF Like Growth Factor; HGF, Hepatocyte Growth Factor; HMG, High Mobility Group; HSP, Heat Shock Protein; IL, Interleukin; IP3, Inositol triphosphate; KLF, Krüppel-like Factor; LDL, Low-Density Lipoprotein; LEF, Lymphoid Enhancer Factor; LIF, Leukemia Inhibitory Factor; LIFR, Leukemia Inhibitory Factor Receptor; LRP, LDL Receptor-related Protein; MAPK, Mitogen Activated Protein Kinase; MC1R, Melanocortin 1 Receptor; MEK, MAPK kinase; MITF, Microphthalmia-Associated Transcription Factor; MMP, Matrix Metalloproteinase; MSH, Melanocyte Stimulating Hormone; nAChR, nicotinic Acetylcholine Receptor; NC-XRE, Nonconsensus Xenobiotic Response Element; p21Cip1 (Cyclin Dependent Kinase Inhibitor); PAH, Polycyclic Aromatic hydrocarbon; PAX, Paired Box; PCB, Polychlorinated Biphenyls; PI3K, Phosphoinositide 3-Kinase; PK, Protein Kinase; PM, Particulate Matter; POP, Persistent Organic Pollutant; PGE, Prostaglandin E; PGF, Prostaglandin F; Raf, Rapidly Accelerated Fibrosarcoma; Ras, Rat Sarcoma; RelB, RELB Proto-Oncogene, NF-kB Subunit); RebBARE, RelB Aryl hydrocarbon Receptor Response Element; ROS, Reactive Oxygen Species; SCF, Stem Cell Factor; SLF, Steel Factor; SOX, SRY-related HMG box; STAT, Signal Transducer and Activator of Transcription; TCDD, 2,3,7,8-tetrachlorodibenzo-p-dioxin; TCF, T-Cell Factor; TF, Transcription Factor; TYR, Tyrosinase; TYRP-1, Tyrosinase Related Protein 1; Wnt, Wingless-related integration site; XAP2, hepatitis B virus X-associated Protein (also known as AIP, Aryl hydrocarbon Receptor Interacting Protein); XRE, Xenobiotic Response Element. Created with BioRender.com.

Melanin biosynthesis is tightly regulated by three melanogenic enzymes, Tyrosinase (TYR), Tyrosinase-related protein 1 (TYRP-1), and Dopachrome Tautomerase (DCT, also referred to as Tyrosinase-related protein 2 - TRP-2), located in melanosomes (Basrur et al., 2003). Tyrosinase is a key enzyme of the melanogenic pathway and primarily responsible for melanin biosynthesis. It catalyzes the oxidation of L-Tyrosine to L-DOPA which subsequently is oxidized to L-DOPAquinone that ultimately produces DOPAchrome, which is further converted into 5,6-dihidroxyindole-2carboxylic acid (DHICA) by DCT. TYRP-1 can oxidize DHICA to indole-5-6quinone carboxylic acid, and further to DHI-melanin (Figure 1—Melanin Synthesis); the DHI and DHICA moieties are part of eumelanin. Pheomelanin synthesis is based on the production of Cysteinyl-DOPA conjugates from DOPAquinone and consists of benzothiazine and benzothiazole groups. TYRP-1 plays a key role in the correct trafficking of TYR to melanosomes (Toyofuku et al., 2001), while DCT is involved in crucial detoxification processes within melanosomes (Milac and Negroiu, 2018). Activation of TYR, TYRP-1, and DCT is controlled by Microphthalmia Transcription Factor (MITF), a key transcriptional regulator of the pathway (D’Mello et al., 2016), which has been subjected to detailed studies looking into mechanisms of hyperpigmentation (Figure 1).

There are two types of skin pigmentation: constitutive and facultative. The constitutive skin color is determined genetically, is not directly affected by sun exposure (or other factors) and has photo-protective properties (Ostrowski and Fisher, 2019). Facultative skin color (tan) is induced by UV exposure, hormonal changes, disease or other stress factors (Ostrowski and Fisher, 2019). Dark skin was shown to have large and numerous melanosomes that are individually bound within a membrane compared to lighter skin, which traditionally produces less melanin and contains smaller melanosomes (Alaluf et al., 2002) aggregated and bound in groups as complexes (Thong et al., 2003; Coelho et al., 2009). These specific characteristics generate the tremendous variation of skin tones in relation to ethnicity and response to toxicants (pollutants) and to products designed to counteract their deleterious effects on skin pigmentation. The complexity of pollutants in terms of composition needs to be superimposed on the melanogenic pathway and its governing factors in order to understand the impacts and to address them at the cellular and molecular level.

## 3 Intracellular signaling pathways crosstalk: impacts of air pollution and tobacco smoke

The regulation of the complex melanogenesis pathway involves a multitude of genes, signaling systems and transcription factors which control the process at various levels, from the melanoblasts development to melanocyte survival, differentiation, and melanin production. In the following sections we will focus on several signaling pathways that govern the melanin synthesis, some established and others that are speculative to a degree. Many pathways have been historically demonstrated primarily based on the impact of UV on skin pigmentation. Even though UV is not the subject of this manuscript, we will use the pathways it impacts to evaluate the crosstalk with others that are affected by air pollution and tobacco.

### 3.1 Mitogen-Activated Protein Kinase (MAPK) signaling pathway and AhR pathway

MAPK signaling plays a key role in cellular proliferation, cell metabolism, and inflammation. It is also known to regulate the transcription of cyclic adenosine monophosphate (cAMP) responsive-element binding protein (CREB) (Estrada et al., 2022) and MITF, which is the master regulator of the melanogenic enzymes in charge of pigments synthesis (D’Mello et al., 2016). Of the multiple promoters of MITF gene, M promoter is selectively used in melanocytes and targeted by several transcriptional factors that impact the melanogenic pathway: paired box 3 (PAX3) protein, SRY-related High Mobility Group (HMG) box 9 and 10 (SOX9, SOX10), lymphoid enhancer factor 1 (LEF-1), one cut domain 2 (ONECUT-2), MITF itself (Steingrímsson et al., 2004; Passeron et al., 2007), and CREB. Through a ligand-receptor system, melanocytes respond to increased levels of hepatocyte growth factor (HGF), basic fibroblast growth factor (bFGF), steel factor (SLF/SCF), leukemia inhibitory factor (LIF) and granulocyte-macrophage colony-stimulating factor (GM-CSF) produced by keratinocytes following UV exposure. They ultimately activate the MAPK pathway (Figure 1), thereby phosphorylating MITF and upregulating the expression of TYR, TYRP-1 and DCT (Yarden et al., 1987; Bonaventure et al., 2013).

Of the signaling systems that act upon the MAPK cascade, the one governed by SLF/SCF/c-KIT plays a key role in melanocyte homeostasis. c-KIT has functional xenobiotic-responsive elements (XREs) in its promoter, which are recognized by the Aryl hydrocarbon Receptor (AhR) (Jux et al., 2011), the key player of one of the signaling pathways pollutants act upon, subsequently impacting skin pigmentation. The MAPK-AhR connection is one of the many cross talking points discussed in this part of the manuscript. For simplicity, the direct connection between c-KIT and AhR pathway is not marked on Figure 1.

AhR is a highly conserved cytosolic transcription factor activated by a broad variety of exogenous and endogenous ligands (Nebert, 2017; Rothhammer and Quintana, 2019). In the absence of a ligand, AhR exists in an inactive/latent state as part of a cytosolic multi-protein complex, consisting of two heat-shock protein 90 molecules (HSP90), immunophilin-like hepatitis B virus X-associated Protein (XAP)2, the co-chaperone protein p23, and pp60src (Nebert, 2017). Activation of the receptor following the interaction with a ligand follows either a canonical or a non-canonical pathway, as further explained.

In the canonical signaling pathway, binding of a ligand to the AhR induces conformational changes that facilitate the nuclear translocation of the AhR-ligand complex, releasing hsp90, XAP2 and p23 (Salminen, 2022). AhR subsequently dimerizes with its partner, a structurally related nuclear protein, aryl hydrocarbon receptor nuclear translocator (ARNT) (Salminen, 2022). The complex binding to XREs induces the transcription of genes involved in the response to toxicants such as those encoding for both phase I and phase II xenobiotic metabolism enzymes [cytochromes P450 (CYP) 1A1, CYP1A2, CYP1B1 and UDP glucuronosyltransferase 1 family polypeptide A6 (UGT1A6)] (Ikuta et al., 2009). The activity of these enzymes can be deleterious because they generate reactive oxygen species (ROS). The contribution of ROS to melanin production is discussed in Section 3.5 as one key cross talking point between several intracellular pathways affecting melanin production. As the canonical pathway unfolds, the dissociated pp60src activates the epidermal growth factor receptor (EGFR) and induces its internalization and nuclear translocation (Agostinis et al., 2007). As a result, the downstream MAPK signal transduction is activated (Noakes, 2015), with direct impact on skin pigmentation (Figure 1). This also results in the transcriptional induction of cyclooxygenase-2 (COX-2) which is responsible for the production of Prostaglandin E2 (PGE2) (Matsumura, 2009), a key player in two complex pathways that crosstalk with the melanogenesis pathway: PGE2 induces downstream inflammatory events that lead to hyperpigmentation (as discussed in Section 3.6) and acts directly on the Protein Kinase C (PKC) pathway (Section 3.3).

As part of the non-canonical AhR signaling pathway, AhR binds to non-consensus XRE (NC-XRE) sites (Huang and Elferink, 2012) that share marked homology with the DNA binding sequence of the Krüppel-like factors (KLFs) family (Wilson et al., 2013). Of this family, KLF6 regulates expression of the p21Cip1 cyclin-dependent kinase inhibitor (Bieker, 2001), which is known to lead to ROS accumulation (crosstalk). RelB gene was shown to mediate another non-canonical pathway by interaction with AhR and binding to an unrecognized genomic RelB/AhR responsive element (RelBAhRE) (Vogel et al., 2007). As a result, production of inflammatory markers was shown to stimulate melanin production (Khmaladze et al., 2020; Fernández-Martos et al., 2021; Nkengne et al., 2021; Moon et al., 2022 - detailed in Table 1), another cross talking point in the complex pathways network governing melanogenesis (see Section 3.6).

**Table 1:** Summary of *in vitro*, *ex vivo*, *in vivo* and clinical studies reporting on the impact of pollution and tobacco on skin pigmentation, including effects of ingredients or products with anti-pollution or anti-tobacco-induced hyperpigmentation activity.

Stress factor category	Study details						
	Reference	Test System Type/Species/Model	Dose/measurement	Length of treatment	Endpoint(s)	Possible mechanism(s)^1^	Conclusions
PM	Vierkötter et al. (2010)	Clinical cross-sectional study	Average level of traffic-related particle emission:	Original SALIA study: 1985-1994	- Skin measurements (SCINEXA)	Oxidative stress	- Significant increase of pigment spots following exposure to traffic-related PM.
		400 European Caucasian women (survivors of the original SALIA study)	899.9 kga^-1^km^-2^ in Ruhr area	Follow-up study included in the manuscript: 2008-2009			
			225.7 kga^-1^km^-2^ in Borken area				
	Peng et al. (2017)	Clinical cross-sectional study	Average PM2.5:	10 years of residency in the area of interest	- Skin measurements (SCINEXA)	Oxidative stress	- PM2.5 was significantly associated with senile lentigines (pigmentation spots) on cheeks and hands.
		400 Chinese women	168.13 µg/m^3^ in Xuanwumen				
			63.56 µg/m^3^ in Yanqing county				
	Flament et al. (2018)	Clinical	AQI evaluated for 270 successive days in 2016	15 years or exposure to air pollution in their respective region	- Pictures: wrinkles, skin texture, pigmentation disorders (10 signs), other skin features such as eye bags, sebaceous pores, skin redness	NA	- Clinical severity of three signs related to pigmentation was found strongly and significantly enhanced by regular exposure to severe chronic urban pollution.
		Two cohorts of 102 Chinese women each from Baoding (highly polluted city) and Dalian (less pooluted city)					
	Krutmann et al. (2020)	*Ex vivo* ^2^	DEP (concentration not provided in the abstract of the poster; poster not available)	Not provided in the abstract of the poster; poster not available	- Melanin content	Oxidative stress	- Increased melanin content and number of melanin-positive cells induced by DEPs.
		Human skin model			- Histology (Fontana-Masson for melanin)		- Increased expression of genes involved in melanin synthesis and MMP-1 (wrinkle formation).
					- Gene expression (*MMP1*)		
	Suo et al. (2020)	*In vitro* cell-based	PM2.5 (0-200µg/mL)	24 hours	- Viability	- Oxidative stress	- PM2.5 exposure decreased cell viability (dose dependent).
		Human keratinocytes (HaCaT cells)			- STR profiling	- Apoptosis via mitochondrial pathway	- PM2.5 exposure inhibited SCF and bFGF secretions (dose dependent).
					- SCF/bFGF secretion (ELISA)		
		*In vitro* cell-based	PM2.5 (0-200 µg/mL)	24 hours	- Viability		- PM2.5 exposure inhibited melanin content and TYR activity (dose dependent).
		Human melanocytes (PIG1 and PIG3V - immortalized)			- Melanin content		- Exposure decreased viability (dose dependent) and increased apoptosis (possibly through mitochondrial pathway).
					- TYR activity		- Melanocyte migration decreased (dose dependent).
					- Cell apoptosis		- PM2.5 exposure suppressed SOD/GSH-Px and enhanced MDA (dose dependent), suggesting oxidative stress response eliciting apoptosis.
					- Cell migration		
					- MDA/SOD/GSH-Px levels		
					- Nuclear protein isolation		
					- Western blot		
	Yang et al. (2020)	*In vitro* cell-based	PM2.5 (50-200 µg/mL)	Not provided	- Viability	ROS-mediated	- Cell viability was not affected.
					- ROS		- PM2.5 increased melanin content (dose dependent) in addition to increasing levels of ROS.
		Melanocytes (species not provided)			- Melanin content		- PM2.5 changed broadly melanocyte gene expression.
					- Whole-genome RNA sequencing		
	Ahn et al. (2022)	*In vivo*	PM (1648a) 20 µg/cm^2^	4 weeks, 5x/week	- Fontana-Masson staining	- Stimulation of melanogenesis-related proteins.	- PM-induced pigmentation *in vivo* as determined by melanin index.
		C57BL6 mice			- Melanin index	- ER stress response (IRE1α mediated)	- Higher melanin contents were observed in the PM-exposed mouse tail skin compared to the vehicle-exposed mouse tail skin.
					- Immunofluorescence staining (CRT)	- AhR	- CRT expression was induced by PM.
		*In vitro* cell-based	PM (5; 10; 20; 50; 100 µg/cm^2^)	4 days	- Cell viability (MTT assay)		- PM concentrations of 50 and 100 µg/cm^2^ induced cytotoxicity.
		Normal human primary epidermal melanocytes	ER stress inhibitor 4-phenyl butyric acid	48 hours (for TYR enzymatic activity endpoint)	- TYR activity		- Treatment with 5 µg/cm^2^ increased the concentration of cAMP and protein levels of Pmel17/gp100, leading to melanin production.
					- Western blotting (MITF, Pmel17/gp100, XBP-1, phosphorylated CAMKII, phosphorylated CREB, CRT, phosphorylated IRE1α, phosphorylated PERK, TYR, GRP94, GRP78, CYP1A1, ATF6)		- PM upregulated the levels of ER-resident *GRP94, GRP78, CRT* mRNAs and proteins.
					- Melanin content		- PM induced CRT expression.
					- RNA sequencing		- In CRT-silenced melanocytes, PM treatment did not increase TYR expression. In melanocytes transfected with scrambled small interfering RNA, PM treatment increased TYR expression.
							- Inhibition of IRE1a (by pre-treatment of cells with STF083010) attenuated PM-induced melanin production.
							- PM enhanced expression of CYP1A1. BaP^2^ (0.125; 0.25; 0.5; 1; 2; 5; 10 µM) also induced CYP1A1.
		*Ex vivo*	PM (20 µg/cm^2^)	5 days, treatment on days 1 and 3	- Fontana-Masson staining		- Pigmentation was induced by PM in the *ex vivo* skin cultures.
		Skin organ culture (abdominal skin, Asia, phototype III-IV)			- Confocal microscopy (IRE1α, MITF, CRT)		- PM-exposed human skin with tape stripping displayed a relatively higher pigmented area than the PM-exposed human skin without stripping, which also showed increased pigmentation.
							- Inhibition of IRE1α (by pre-treatment of cells with STF083010) attenuated PM-induced melanin production.
							- PM-induced melanin was reduced by pre-treatment with the IRE1α inhibitor (STF083010).
	Grether-Beck et al. (2021)	*Ex vivo* ^2^	DEP (6 and 40 µg cm^2^)	6 and 9 days	- Viability	Oxidative stress	- Treatment increased melanin content without decrease in viability.
		Human skin	Pre-treatment with anti-oxidants cocktail (vitamin C, vitamin E and CEF): only for the clinical study with 20 participants^4^		- Antioxidant levels		- Treatment increased TYR expression and oxidative DNA damage.
					- Melanin content		
		Clinical		9 days	- Skin color (chromametry)		- Repetitive application of DEP increased constitutive skin pigmentation in a time-dependent manner.
		Düsseldorf pollution patch test (76 participants)			- Melanin index (color meter)		
		Clinical Vehicle-controlled, randomized, intra-individual comparative double-blinded study (20 participants)		14 days			- Increase in melanin staining and in the transcriptional expression for genes important for the *de novo* melanin synthesis such as *POMC, EDN1, MITF, TYR, TYRP1, MLANA,* and *PMEL.*
							- Pre-treatment with antioxidants inhibited DEP-induced pigmentation.
	Nkengne et al. (2021)	Clinical multicenter cohort study	Air pollution evaluated by CAQI in Guangzhou and Zhanjiang	5 years of residency in the area of interest	- Melanin index	Inflammation	- Redness was lower while yellowness was higher with high level of air pollution (specific to Guangzhou area).
		203 Chinese women			- Hb index		- Melanin index was higher on eyelid with high pollution levels (specific to Guangzhou region).
	Shi et al. (2021)	*In vitro* cell-based	PM2.5 (0-500 µg/mL)	24, 48 and 72 hours	- Viability	AhR/MAPK pathway	- No cytotoxic damage <200 µg/mL.
		Human Epidermal Melanocytes	PM2.5 (0-200 µg/mL)	48 and 72 hours	- Melanin content		- PM2.5 increased melanin content and TYR activity (dose dependent).
					- TYR activity		
					- PCR		- PM2.5 increased melanogenesis-related protein expression (not dose dependent).
					- Western blot (72 hours only)		
		*In vitro* cell-based	PM2.5 (0-200 mg/mL)	24 hours	- ELISA		- PM2.5 increased α-MSH production by keratinocyte which further induced hyperpigmentation (dose-dependent).
		Human keratinocytes (HaCaT cells)					- PM2.5 increased expression of *TYRP1, TYR, MITF* genes in supernatant of HaCaT cells.
		*Ex vivo*	PM2.5 (100 µg/mL)	7 days (daily treatment)	- Histology (staining for melanin)		- PM2.5 increased melanin content.
		Human skin					- The ratio of the pigmented area to the total epidermal area was greater in the PM2.5-treated skin samples compared to controls.
		*In vivo*	PM2.5 (0, 50, 100 and 200 µg/mL)	7 days (daily treatment)	- Fontana-Masson staining of tissue sections for melanin analysis		- PM2.5 (100 µg/ml) increased melanin content in a time dependent manner.
		Wild-type TU zebrafish embryos					- Pigment area density was more than two-folds greater in PM2.5-exposed zebrafish compared to the control.
	Yang et al. (2022)	*In vitro* cell-based	PM2.5 (**3.13; 6.25; 12.50; 25; 50**; 100; 200; 400 µg/ml)	24 hours	- Viability	- Melanin synthesis through keratinocyte-melanocyte route (epidermal-melanin unit cross-talk)	- Viability of cells treated with PM2.5 concentrations ≤12.5 µg/ml and BaP ≤5 µmol/l was not impacted.
		Normal human epidermal keratinocytes	BaP (0.5; **1; 2.5; 3.5; 5; 10**; 20; 50 µmol/l)		- Cell morphology (for the cells treated with the concentrations presented in bold)		
		*In vitro* cell-based	PM2.5 (3.13; 6.25; 12.50; 25; 50; 100; 200; 400 µg/ml)	24 hours	- Viability-		- Viability: see note above for keratinocytes culture.
		Normal human melanocytes	BaP (0.5; 1; 2.5; 3.5; 5; 10; 20; 50 µmol/l)				
		*In vitro*	PM2.5 (7.5 and 12.5 µg/ml; 10 µl dosing volume)	7 days	- Melanin content		- No changes in morphology.
					- Melanin distribution		- Tissues treated with 12.5 µg/ml PM2.5 and 5 µmol/l BaP became darker than the controls, the melanin content increased and the melanin particles increased mainly in the lower parts of the sections.
		Reconstructed human epidermis model (MelaKutis®, BioCell Biotechnology, China)	BaP (3; 5 µmol/l; 10 µl dosing volume)		- H&E staining		
					- Fontana-Masson staining		
					- Brightness		
PAH	Krutmann et al. (2008)	*In vitro* cell-based	TCDD and FICZ (dose not available - details presented herein were retrieved from abstract of poster; poster not available)	Not available (details presented herein were retrieved from abstract of poster; poster not available)	- Gene and protein expression (AhR, TYR, TYRP-1)	AhR	- Primary murine and human melanocytes express AhR at the mRNA and protein level.
		Primary murine and human melanocytes			- CYP4501A1/1B1 assay		- TCDD induced CYP4501A1/1B1 which is a marker of the AhR signaling pathway. This induction was abrogated in murine melanocytes by antagonist MNF.
					- Melanin assay		- TCDD and FICZ induced TYR enzymatic activity in human primary melanocytes, a process AhR dependent (inhibited by MNF).
							- Triggers of AhR induced mRNA expression of Tyr and Tyrp-1 in murine melanocyte and subsequently the melanin production.
		*In vivo*			- Melanin assay		- Melanocyte density and melanin content were greatly reduced in the skin of AhR -/- mice compared to wild-type litter mates.
		AhR knockout (AhR-/-) mice			- Melanocyte density		
	Luecke et al. (2010)	*In vitro* cell-based	TCDD (10 nM)	3 days	- Melanin assay	AhR (not modulated via the PKC-β)	- Normal human melanocytes expressed AhR.
		Normal human melanocytes	MNF (1 µM)		- RT-PCR (*AhR, AhRR, CYP1A1, CYP1B1, TYR, DCT*)		- Activation of the melanocytes by 10 nM TCDD resulted in the activation of the AhR pathway and the upregulation of CYT1A1 and 1B1 and AhRR. The expression was abolished by co-treatment with the partial antagonist MNF.
					- Western blotting		- Treatment of human melanocytes with TCDD upregulated TYR activity; co-treatment with partial antagonists MNF or α-naphthoflavone abolished the TCDD-induced TYR activity. Melanin production was also elevated by treatment with TCDD. These upregulations are not due to enhanced cell proliferation.
					- Tyrosinase activity assay		
					- Cell viability (WST-8)		
	Abbas et al. (2017)	*In vitro* cell-based	1; 2.5; 5; 10; 25 and 50 µM of BA	24 hour exposure	- MTT assay - % viability compared to vehicle control	AhR	- Concentrations of 1 and 2.5 µM of BA did not induce significant decrease in viability. Dose response present at 5-50 µM (used for selecting 5, 10 and 25 µM).
		Primary Mouse Melanocytes	5, 10 and 25 µM of BA	24 hour exposure	- Immunofluorescence – staining for AhR		- Increased melanin content after treatment.
			10 nM TCDD (for AhR immunofluorescence, melanin content and PCR endpoints)		- % melanin content compared to vehicle control		- Significant enhancement in *AhR* mRNA expression following exposure to BA (all doses).
					- RT-PCR		- TYR activity was higher compared to the control (dose-dependent for exposure to 10 and 25 µM BA).
					- TYR enzymatic activity		
		*In vivo*	50 mg/kg. b. wt/100 µl acetone, daily (BA)	28 days	- Western Blot Analysis		- Enhanced pigmentation and melanin synthesis determined in mice exposed to BA and TCDD.
		C57BL/6 mice	125 ng/mouse/100 µl acetone, three times/week (TCDD)		- TYR activity in skin lysates		
					- Fontana-Masson staining for melanin		
					- Immunofluorescence - staining for TYR		
	Chan et al. (2021)	*In vitro* cell-based	PAH: IcdP (250 nM), BaP (100 nM), BbF (250 nM), DBA (250 nM)	72 hours	- Cytokine/chemokine profiling: IFNγ, TNFα, IL-8, IL-6, GM-CSF, IL-1β, IL-10	- Activation of T cells (BaP and BbF)	- IFNγ was upregulated by BaP and BfB.
						- Monocytes activation (DBA)	- DBA stimulated production of TNFα and IL-8.
		PMBCs			- PCR array	- AhR	- AhR-related genes were significantly upregulated in BaP-treated PBMCs.
						- Oxidative stress	
		*In vitro* cell-based	Pre-treatment with vitamin E (50 µM), vitamin C (100 µM), sulforaphane (10 µM) dexamethasone (0.1 µM or 1 µM) followed by conditioned medium from BaP-exposed PBMCs^5^	24 hours pre-treatment followed by 24 hours treatment	- DDR quantification	- Oxidative DNA damage	- DSB increased in keratinocytes following the exposure to the conditioned medium (p53-dependent).
		Human primary keratinocytes			- Cell viability (MTT assay)	- AhR	- Levels of 8OHdG were significantly upregulated in the treated keratinocytes.
					- DNA extraction for 8OHdG quantification	- Oxidative stress	- AhR-related genes were significantly upregulated in keratinocytes treated with the conditioned media.
					- Media collected for PBMCs quantification		- All three antioxidants used for pre-treatment reduced DSB in keratinocytes.
							- POMC production by keratinocytes was significantly reduced by antioxidants that may act as corrective/preventive solutions for pollution-induced pigmentation disorders.
		*In vitro* cell-based/3D model	Pre-treatment with vitamin E (50 µM) and vitamin C (2 mM)	7 days treatment (alternate days)	- Color measurement (spectrocolorimeter)	- Melanin transfer mediated by BaP	- RHPE exposed to PBMCs had darker color compared to the models exposed to the control (DMSO) or BaP alone.
		PMBCs co-cultured with RHPE (phototype IV, day 11, Episkin, France)	Treatment with or without BaP for 7 days	Harvest at 17 days	- Fontana-Masson staining for melanin		- The Fontana-Masson staining showed a significant increase in the total melanin content in the tissues treated with PBMCs and BaP compared to the tissues treated with PBMCs and DMSO.
					- DOPA staining		- Melanin levels in the upper layer of the tissues were significantly increased in the tissues co-cultured with PBMCs and treated with BAP as compared to the tissues co-cultured with PBMCs and treated with either DMSO or BaP alone.
		*In vitro* cell-based	PAHs alone (see concentrations above, in PBMCs section)	72 hours	- Melanin synthesis	- PAHs and BaP-activated PBMCs do not directly stimulate melanogenesis	- No increase in melanin content or melanogenesis-associated gene expression were observed.
		Human primary melanocytes	BAP-conditioned medium from BaP-activated PBMCs		- Gene expression (*MITF, TYR*)		
PCBs	Leijs et al. (2018)	Clinical medical surveillance program HELPcB (92 participants former workers from a transformer recycling company)	Potential exposure to PCBs and/or dioxin (polychlorinated dibenzon-*p*-dioxin/polychlorinated dibenzo-*p*-furan) levels	Prior exposure to PCB and dioxins; evaluation performed in 2014	- Questionnaire on exposure to toxicants	- AhR (CYP1A1 induction)	- The probability of hyperpigmentation on the skin was significantly higher in workers with higher sum of total PCBs-, dioxin like-PCBs and dioxin levels.
					- Plasma analysis for PCBs and dioxins	- Oxidative stress	
Anti-pollution products	Milani et al. (2019)	Clinical, explanatory, experimental, prospective, pilot, single-blinded, controlled study (20 Italian women)	*Deschampsia antartica*/Ferulic acid/vitamin C (DAE/FC/VC) serum	28 days (twice daily application on the entire face)	- TEWL	- Oxidative stress	- TEWL was reduced with treatment, dark spots were reduced and SQOOH/SQ ratio improved after 28 days of treatment.
					- Skin color	- AhR	
					- SQOOH/SQ skin ratio		
	Khmaladze et al. (2020)	*In vitro* cell-based	Active D (acetyl dipeptide 1 cetyl ester)	24 hour exposure	- Cell viability	- Oxidative stress	- Upregulation of important skin barrier function genes such as *AQP3, FLG*, *Caspase14, Keratin10* compared to controls.
		Primary human keratinocytes			- RT-PCR (barrier function)		
		*In vitro* cell-based	DPM, 1650b (10 µg/ml)^6^	24 hours	- Cell viability	- Inflammation	- *See notes on spheroids*.
		Immortalized keratinocytes (HaCaT)			- RT-PCR (barrier function)		
		*In vitro* cell-based	Pre-treatment with active A (0.5% or 1%) (diglucosyl gallic acid) before the addition of the media from the keratinocytes treated with DPM, 1650b (10 µg/ml)	1 hour pre-treatment followed by 24 hour exposure in media from DPM-treated keratinocytes	- Cell viability	- Barrier dysfunction	- Significant inhibition of DPM-induced MMP1 in the cells treated with formulations compared to controls.
		Primary human dermal fibroblasts		For glycation assay: induction with 0.5 mmol/l glyoxyal for 36 hours followed by 72 hours in presence of active C (1 mmol/l) and active D (6 µg/ml) or a combination of both	- RT-PCR (dermal gene expression)		- Treatment with actives C and D or their combination resulted in a significant upregulation of genes involved in skin wrinkling: *LOXL1, COL1A, DCN, FBN1.*
				For proteasome activity: pre-treatment with active C (niacinamide) and active D (acetyl dipeptide 1 cetyl ester) for 3 hours	- MMP1 ELISA		- Active C was a very potent anti-glycant and active D had an intermediate activity.
					- Immunofluorescence (CML adducts)		
					- Proteasome activity		
		*In vitro* cell-based	Active A (diglucosyl gallic acid)	5 days	- Cell viability		- Active B alone or the combination of all treatments showed anti-melanogenic activity at gene level (*MITF*).
		Primary human epidermal dark pigmented melanocytes	Active B (caprylic/capric triglyceride, diacetyl boldine)		- RT-PCR (expression of MITF)		- Melanin inhibition by active B and by the combination of all treatments.
			Active C (niacinamide)		- Melanin assay		
		*In vitro* cell-based	Actives A, B, C	5 days	- Melanin assay		- Active B alone or the combination of all tested actives were the most efficient anti-melanogenic treatments.
		Co-culture: primary human epidermal dark pigmented melanocytes and immortalized keratinocytes (HaCaT) (considered 3D spheroid model)					
		*Ex vivo*	Formulation complex: oil in water emulsion blended with actives A (1%), B ([4%], caprylic/capric triglyceride [qsp 100], diacetyl blodine [∼1000 ppm]), C (5%), dD (4%)	1 hour pre-treatment with formulations followed by 5 hours topical treatment of DPM (0.5% in propylene glycol)	- RT-PCR for CYP1A1		- Significant inhibition of DPM-induced *CYP1A1* gene in test system treated with formulations compared to placebo.
			Formulation complex (*See breakdown above*)	24 hours pre-treatment with formulations followed by 24 hour topical treatment with DPM (0.5% in propylene glycol)	- MMP1 ELISA		- Significant inhibition of DPM-induced MMP1 in test system treated with formulations compared to placebo.
		Human skin (8-10 mm full thickness)			- IL-6 ELISA		- DPM-induced IL-6 cytokine inhibition with formulation complex compared to placebo.
			Formulation complex (*see breakdown above*)	72 hours (applied topically daily)	- Histology (for FLG, FBN1)		- Significant upregulation of FLG compared to placebo.
	Krutmann et al. (2020)	*Ex vivo*	Anti-oxidant cocktail containing vitamins C and E, neohesperidine and maritime pine polyphenols as active ingredients (Liftactive Cure) applied after stimulation with DEP (*See notes on the same reference listed above, in the “DEPs”subsection of the “PM” section*)	Not provided in the abstract of the poster; poster not available	- Melanin content	Oxidative stress	- DEP-induced hyperpigmentation mediated by oxidative stress was reduced by the product tested.
		Human skin model			- Histology (Fontana-Masson for melanin)		- Gene expression of MMP-1 was also reduced by Liftactive Cure.
					- Gene expression (MMP1)		
	Fernández-Martos et al. (2021)	*Ex vivo*	Extract of *Deschampsia Antarctica* (2.5 mg/ml) (Edafence)	24 hours exposure to all toxicants except for TCDD (3 hours)	- H&E	- AhR	- Pollutant exposure led to extensive epidermal-dermal detachment, appearance of inflammatory infiltrate between dermis and epidermis and slight edema in the epidermis. All of these morphological changes were prevented by Edafence pre-treatment but no difference for hyperpigmentation was observed.
		Human Skin Organotypic Culture	Toxicants: 9 mM NaAsO_2_ (As(III), arsenic), 0,3 mM CdCl_2_ (Cd(II), Cadmium), 0.5 mM CrO_3_ and CrCl_3_, at equal concentrations, (Cr(III/VI), Chromium)		- Immunohistochemistry (E-cadherin, AhR)	- Inflammation	- Depending on the pollutant, expression of E-cadherin receptor was downregulated partially or completely (avoided with Edafence pre-treatment).
			10 nM TCDD		- Gene expression analysis (oxidative stress)		- Pre-treatment with Edafence prevented AhR overexpression induced by pollutants.
			Tobacco smoke extract infusions (100%, 20 cigarettes in 20 ml of DMEM)^7^	Pre-treatment with the extract for 24 hours before exposure to toxic compounds	- Proliferation markers		- Cytokine (IL-6, IL-8) response was promoted with pre-treatment with Edafence.
					- Cell death assays		- Overall, Edafence counterbalanced the deleterious effects of toxicants and triggered activation of key genes involved in the redox system and pro-inflammatory/would healing response in the skin.
							- Tobacco smoke modulated the expression and activity of AhR-regulated genes and AhR-dependent drug metabolizing enzymes.
	Moon et al. (2022)	*In vitro* cell-based	PM10 (50-400 µg/mL)	72 hours	- Cell viability	- Oxidative stress	- No change in pigmentation was observed after 3 days of treatment.
		Human Epidermal Melanocytes			- Melanin content		
		*In vitro* cell-based	PM10 (100 µg/mL) and 50 µg/mL of saponins of Korean red ginseng for intracellular ROS assay	24 hours (intracellular ROS assay)	- Cell viability		- PM treatment decreased viability (dose dependent). Concomitant treatment with saponins promoted cell viability.
		Human Epidermal Keratinocytes	Pre-treatment with 50 µg/ml saponin, 50 µg/ml nonsaponin or 1 mM *N*-acetyl-L-cysteine	1 hour pre-treatment with saponin, nonsaponin or *N*-acetyl-L-cysteine followed by treatment with 100 µg/ml PM10 for 3 hours	- Gene expression		- Saponin treatment also decreased intracellular oxidative stress from PM10.
					- Intracellular ROS assays		- Expression of pro-inflammatory cytokines was mitigated by saponin treatment.
					- MMP-3 assay		- Expression of SCF and ET-1 increased with PM treatment but lowered with saponin treatment.
					- RT-PCR	- MITF	- The mRNA expression of *MMP-3* increased dramatically after exposure to PM10, but this change was effectively attenuated by concomitant treatment with saponins (50 µg/ml).
					- Cytokine assays (IL-1α, IL-1β, IL-8)		- Exposure of keratinocytes to 100 µg/ml PM10 led to increases in cytokine expression and level of leptin that were mitigated by concomitant treatment with 50 µg/ml saponins.
					- SCF, ET-1 assay		- Induction of IL-6 by LPS was attenuated significantly by treatment with saponins (100 µg/ml).
		*In vitro* cell-based	PM10 (50-400 µg/mL) and treatment with saponins (50 and 100 µg/ml)	24 hours	- Cell viability		- Increase in melanin content (dose dependent) that was mitigated with saponin concomitant treatment (dose dependent).
		Co-culture of keratinocytes and melanocytes (1:1)			- Melanin content		- Expression of MITF and TYR was induced after exposure to PM10 (100 µg/ml) but was mitigated by treatment with 50 µg/ml saponin treatment.
					- Gene expression (MITF, TYR)	- Inflammation	
		*In vitro* cell-based	PM10 (100 µg/ml)	Not provided	- Cytokine assays (IL-1α, TNF-α)		- A significant increase in the mRNA expression of IL-1α and TNF-α was noted, which was reversed by concomitant treatment with 50 µg/ml saponins.
		Murine macrophages RAW264.7	50 µg/ml saponins				
		*Ex vivo*	PM10	6 days	- Melanin content (histological staining)		- Suprabasal melanin content was increased by exposure to PM10.
		Human skin model					
Tobacco-related fractions	King et al. (2009)	Clinical study (147 African American humans)	Current smokers (exposure to tobacco)	3 months (collection of data)	- Melanin Index for facultative and constitutive melanin (reflectometer)	Nicotine affinity for melanin-containing tissues	- Facultative melanin significantly related to CPD. Constitutive melanin was not significantly related to CPD but both were significantly related to duration of smoking.
					- Survey		- Salivary levels of cotinine were significantly related to facultative melanin but not constitutive melanin.
					- EIA (cotinine assessed in saliva)		
	Serri et al. (2010)	Clinical study (64 Caucasian women)	Smokers enrolled in a cessation program (February to November 2007)	Evaluations at the start of the program and at 3, 6, and 9 months	- Lines	NA	- At the start of the program, the participants had presented with an average biological age of 9 years old then their chronological age.
					- Vascular and pigmentation state		- At the end of the program, an average reduction of about 13 years in the biological age of the participants’ skin was found.
					- Elasticity		
					- Brightness		
					- Texture of the skin		
	Cho et al. (2012)	Clinical study (34 South Korean men)	No exposure (smoking cessation program)	1 and 4 weeks after smoking cessation	- Melanin index (Mexameter)	- Oxidative stress	- Melanin index on majority of face and abdomen sites decreased 1 week and 4 weeks after smoking cessation (baseline) to a similar level.
					- Erythema index on the face and lower abdomen	- Inflammation	- Erythema index on majority of face and abdomen sites decreased 1 week and 4 weeks after smoking cessation.
						- Hb capacity to delivery oxygen	- Upward shift in Hb distribution curve resulted in a reduction in the ability to delivery oxygen to the tissues.
	Ishiwata et al. (2013)	Clinical study (84 Japanese participants)	Smoking cessation treatment (success/failure)	0-12 weeks	- *Stratum corneum* carbonyl protein levels quantification	Oxidative stress (inducing carbonylation and reducing *stratum corneum* transparency)	- A decreased tendency of carbonyl protein in the success group was observed, but was not statistically significant.
					- Skin color measurements (converted to melanin, oxyHb and reduced Hb)		- Skin color tended to increase in lightness and decrease in redness depending on length of treatment and success/failure groups. OxyHb and deoxHb showed decreasing tendency during cessation treatment; no changes in melanin levels were noted.
	Nakamura et al. (2013)	*In vitro* cell-based	Cigarette smoke extracted in PBS	20 days exposure to tobacco smoke extract + UVB exposure every other day	- Pigmentation levels assessed	- Wnt/β-catenin	- Cells cultured with cigarette smoke had normal morphology but increased UV-induced pigmentation.
		Human epidermal melanocytes		32 hours exposure to tobacco smoke extract + UVB irradiation at 24 hours	- PCR (mRNA purification performed at 8 hours after irradiation) - *MITF*, *β-catenin*	- AhR	- Increase in MITF (dose dependent) and β-catenin expression.
			0.2 and 2 µl/mL of smoke extract	72 hours	- AhR expression		- siRNA was used to knockdown the AhR. The tobacco smoke-induced MITF activation of siRNA-treated melanocytes was significantly inhibited, indicating melanocyte activation through AhR.
	Delijewski et al. (2014)	*In vitro* cell-based	Nicotine solution (0.0001 to 10 mM)	21 hours	- Viability	- Epinephrine (augmented while smoking) and cAMP	- Viable cells at 0.0001 to 0.01 mM. The value of EC_50_ was determined to be 2.52 mM.
		Normal Human Melanocytes		24 hours	- Melanin content		- Melanin content increased by nicotine (0.01 and 0.05 mM), decreased at 1.0 mM.
					- TYR activity	- Nicotine affinity to melanin (nicotine adducts formation with melanin’s intermediate, dopaquinone)	- TYR activity increased (0.01 and 0.05 mM), suppressed at 1.0 mM.
					- Enzyme activity (SOD, CAT, GPx)	- Oxidative stress	- SOD, CAT activity and hydrogen peroxide content increased by nicotine and GPx activity decreased (0.05, 0.1, 0.5 and 1.0 mM).
					- Hydrogen peroxide content		
	Tamai et al. (2014)	Clinical study (939 Japanese women)	Smoking status: current, never, and former smokers	2003-2006 (collection of data)	- Skin color measurements (melanin and erythema index) of inner arms and forehead	- Nicotine affinity to melanin	- Melanin significantly higher for current smokers compared to never or former smokers and associated with number of cigarettes smoked daily/number of years smoking. Erythema significantly higher with number of daily cigarettes/number of years smoking. Smokers more likely to have darker skin.
				Life time smoking status: 20 packs		- Hb contribution	
	Nakamura et al. (2016)	*In vitro* cell-based	Tobacco smoke extract (2 µl/ml)	48 hours (exposure followed irradiation: 310 nm, 5 mJ/cm^2^	- RT-PCR for AhR expression	- AhR	- AhR expression increased in cells cultured with tobacco smoke extract.
		NCCmelb4 derived from mouse neural crest cells					
	Liakoni et al. (2019)	Clinical study (44 African-American smokers)	IV infusion of 1.5 mg/kg/min of deuterium-labeled nicotine and cotinine	30 minutes IV (length) samples collected at 10, 20, 30, 45, 90 minutes and 2,3, 4, 6, 8, 12, 16, 23, 47, 71 hours after dosing	- NMR in plasma	NA	- Contradicting results compared to King et al., 2009 likely due to study design differences.
					- Constitutive and facultative melanin index		- The study found no evidence of significant differences in nicotine pharmacokinetics based on melanin index and no evidence of significant correlations between pharmacokinetic parameters of nicotine or cotinine, or tobacco dependence measures and melanin levels.
					- CYP2A6 (hepatic) genotyping		
	Yazdanparastet al. (2019)	Clinical analytical, cross-sectional study (28 participants)	Smoking status: current and non-smokers	NP	- *Stratum corneum* hydration	NA	- Melanin content was higher in smokers but not significant.
					- TEWL		- Current smokers had higher melanin indices than never-smokers and former smokers.
					- pH		
					- Erythema index		
					- Melanin index		
					- Friction value		
					- Skin elasticity measurements		
	Dalrymple et al. (2022)	Clinical study (10 non-smokers)	CS, THP and EC aerosols on the scapula using an exposure chamber	32 cumulative puffs	- Skin color	Oxidative stress	- Skin color darkened, reddened and yellowed with CS exposure.
					- SQ		- SQOOH levels increased with CS exposure, SQ levels remained similar. SQOOH/SQ ratio higher with CS exposure.
					- SQ OOH		- MDA values increased with CS exposure.
					- MDA		- Catalase values comparable between all treatments.
					- CAT levels in skin sebum		

**Abbreviations**: 3D, three-dimensional (when referring to reconstructed tissue models); 8OHdG, 8-hydroxydeoxyguanosine; AhR, Aryl hydrocarbon Receptor; AhRR, Aryl hydrocarbon Receptor Repressor; AQI, Air Quality Index; AQP, Aquaporin; ATF, Activating Transcription Factor; BA, Benzanthrone; BaP, Benzo(a)pyrene; BbF, Benzo(b)fluoranthene; bFGF, basic Fibroblast Growth Factor; cAMP, cyclic Adenosine Monophosphate; CAMKII, Calcium-Calmodulin-dependent Protein Kinase II; CAT, Catalase; CAQI, Comprehensive Air Quality Index; CEF, CE Ferulic®; CML, Carboxy-methyl lysin; COL1a, Collagen Type 1; CREB, cAMP Response Element-Binding Protein; CML, carboxymethyl lysin adducts; CPD, cigarettes smoked per day; CRT, Calreticulin; CS, Cigarette Smoke; CYP, Cytochrome; DBA, Dibenz(a,h)anthracene; DCN, Decorin; DCT, Dopachrome tautomerase; DDR, DNA Damage Responses; DEP, Diesel Exhaust Particles; DMEM, Dulbecco’s Modified Eagle Medium; DMSO, Dimethyl Sulfoxide; DOPA, 3,4-dioxyphenylalanine; DPM, Diesel Particulate Matter; DSB, Double Strand Breaks; EC, Electronic Cigarette; EC_50_, half maximal effective concentration; END-1 (EDN1, ET-1), Endothelin-1; EIA, Enzyme immunoassay; ELISA, Enzyme-linked Immunosorbent Assay; ER, Endoplasmic Reticulum; FBN1, Fibrillin-1; FICZ, 6-formylindolo[3,2-b]carbazole; FLG, Filaggrin; GM-CSF, Granulocyte-Macrophage Colony-Stimulating Factor; GRP, Glucose-regulated Protein; GGSH-pX (GPx), glutathione peroxidase; Hb, Hemoglobin; H&E, Hematoxylin and Eosin; HELPcB, Health Effects in high-Level Exposure to PCB; IcdP, Indeno(1,2,3-cd)pyrene; IFN, Interferon; IL, Interleukin; IRE, Inositol-requiring transmembrane kinase/endoribonuclease; IV, intravenous; LOXL1, Lysyl Osidase-Like 1; LPS, Lypopolysaccharide; MAPK, Mitogen Activated Protein Kinase; MDA, malondialdehyde; MITF, microphthalmia transcription factor; MLANA (also known as MELAN-A or MART-1), Melanoma Antigen Recognized by T-cells; MSH, Melanocyte Stimulating Hormone; MMP, Matrix Metalloproteinase; MNF, 3’methoxy-4’nitroflavone; MTT, 3-(4,5-dimethylthiazol-2-yl)-2,5-diphenyl-2H-tetrazolium bromide; NA, Not Applicable; NMR, Nicotine Metabolic Ratio; NP, Not Provided; PAH, Polycyclic Aromatic hydrocarbon; PBMC, Peripheral Blookd Mononuclear Cells; PBS, Phosphate-Buffered Saline; PCB, Polychlorinated Biphenyls; PERK, Protein Kinase R-like ER Kinase; PKC, Protein Kinase C; PM, Particulate Matter; PMEL (Pmel), Premelanosome protein (also known as gp100); POMC, proopiomelanocortin; RHPE, Reconstructed Human Pigmented Epidermis; ROS, Reactive Oxygen Species; RT-PCR, Reverse Transcription Polymerase Chain Reaction; SCF, Stem Cell Factor; SCINEXA, Score of Intrinsic and Extrinsic Skin Aging; SOD, Superoxide Dismutase; SQ, Squalene; SQOOG, Squalene Monohydroxyperoxide; STR, Short Tandem Repeat (profiling); TCDD, 2,3,7,8-tetrachlorodibenzo-p-dioxin; TEWL, Trans-Epidermal Water Loss; THP, Tobacco heating Product; TNF, Tumor Necrosis Factor; TYR, Tyrosinase; TYRP-1, Tyrosinase related protein 1; UV, Ultraviolet; WST-8, Water-Soluble Tetrazolium-8; Wnt, Wingless-related integration site; XBP, X-box Binding Protein.

**Notes:**

- The list covers most or all studies the authors had access to at the time of writing the manuscript.

- The studies are listed in each section in chronological order and in alphabetical order within the same year (where applicable).

1 Mechanisms either demonstrated by results reported in the manuscripts or inferred based on other cross-referenced studies.

2 Study can be considered also for the section “PAH”.

3 There is a possibility for the test system to be the same between these two studies (the *ex vivo* model).

4 Study can be considered also for the section “Anti-pollution products”.

5 Study can be considered also for the section “Anti-pollution products”.

6 Study can be considered also for the section “PM”, sub-section “DEP” (alongside references Krutmann et al., 2020; Grether-Beck et al., 2021).

7 Study can be considered also for the section “Tobacco-related fractions”.

Originally, AhR was identified as a TCDD-binding receptor protein and was later demonstrated to also recognize benzo[a]pyrene (BaP) (Tsuji et al., 2011; Nakamura et al., 2016), other dioxins, PAHs (Schecter et al., 2006), benzanthrone (BA) (Abbas et al., 2017), and related persistent organic pollutants (POPs) (Nebert, 2017) as ligands leading to hyperpigmentation (Luecke et al., 2010; Jux et al., 2011; Esser et al., 2013). TCDD, BaP, and benzanthrone are also components of tobacco and research by Tsuji et al., 2011; van den Bogaard et al., 2013; Nakamura et al., 2016; Abbas et al., 2017 supported their involvement in stimulation of melanogenesis. Furthermore, AhR also mediates melanoblast to melanocyte maturation and plays wider roles in skin melanogenesis (Esser et al., 2013; Nakamura et al., 2013). The contribution of AhR to melanin production in response to environmental stimuli is further supported by evidence provided by UV exposure as another proof of pollutants using established intracellular pathways leading to hyperpigmentation. For example, genes targeted by AhR were shown to be induced by UVB irradiation (Noakes, 2015). In sum, skin’s exposure to environmental stimuli can activate hyperpigmentation *via* AhR activation and subsequent increase of TYR activity, melanin synthesis, and upregulation of TYRP-1, DCT, and MITF expression (Krutmann et al., 2008; Luecke et al., 2010; Nakamura et al., 2013; Nakamura et al., 2016; Abbas et al., 2017; Leijs et al., 2018; Chan et al., 2021; Shi et al., 2021; Ahn et al., 2022) (Table 1).

### 3.2 Protein Kinase A (PKA) signaling pathway

The activation of adenylate cyclase (AC) upon exposure to UV as primary stimulus or to other factors catalyzes the conversion of ATP to the second messenger cAMP, which attaches to PKA leading to its activation. PKA subsequently phosphorylates CREB that can enhance MITF’s overexpression (Shin et al., 2015), with direct contribution to melanin production. Proteolytic cleavage of the proopiomelanocortin (POMC) protein results in the formation of adrenocorticotropic hormone (ACTH) and alpha-melanocyte-stimulating hormone (α-MSH). Both these factors are produced by keratinocytes in response to UV exposure and act as agonists to melanocortin 1 receptor (MC1R) on melanocytes, thereby increasing cAMP levels (Yamaguchi and Hearing, 2009) (Figure 1). At this point, the PKA and MAPK cascades crosstalk and contribute to melanin production through the common factor CREB (Figure 1). Evidence of tobacco or air pollutants acting directly on this signaling pathway are lacking. Given the common players among the MAPK, PKA and AhR pathways, it is conceivable to believe that the PKA could be chosen by environmental stimuli as direct or indirect port of entry to melanocytes. The only link between the AhR and PKA signaling routes was identified in the study by Bahraman et al., 2021 showing that the increase of α-MSH-induced expression of AhR may be due to the influence of cAMP directly or indirectly *via* the Wingless-related integration site (Wnt)/β-catenin pathway or both pathways. The participation of β-catenin to skin pigmentation in response to stimuli is discussed later (Section 3.4). Last but not least, there is another intersection point that could be of interest in the context discussed herein: between the PKA and adrenergic signaling pathways (Section 3.9).

### 3.3 Protein Kinase C (PKC) signaling pathway

The PKC pathway regulates melanogenesis (Gordon and Gilchrest, 1989) following the binding of Endothelin (EDN) 1 to its receptor (EDNRB) and of PGE2/PGF2 to their respective receptors, EP1/EP3/FP, in response to UV exposure (Park et al., 2006) (Figure 1). After the complex formation, phospholipase C β (PLCβ) is activated and catalyzes the production of inositol triphosphate (IP_3_) and diacylglycerol (DAG). IP_3_ induces the production of cytosolic Ca^2+^ response in melanocyte dendrites (Stanisz et al., 2012; Belote and Simon, 2020), whereas DAG activates PKC, which enhances the expression of MITF directly or indirectly via the MAPK cascade (Serre et al., 2018) (crosstalk) (Figure 1). In addition, the EP3 receptor controls the effects of PGE2 on cAMP in melanocytes that stimulates TYR activity and proliferation (Scott et al., 2004; Scott et al., 2005) (cross talk with PKA signaling pathway - see Section 3.2). The signaling of the fourth receptor of PGE2, EP4, stimulates cAMP production in melanocytes and subsequently TYR and the formation of dendrites (Starner et al., 2010), with direct implications in melanin biosynthesis (for simplicity, not displayed in Figure 1).

PKC usually resides in an inactive state in the cytoplasm but is activated by DAG generated from UV-irradiated cell membranes (Bae-Harboe and Park, 2012). DAG induces PKC translocation from the cytoplasm to membranes where PKC catalyzes the phosphorylation of serine residues on the cytoplasmic domain of TYR, thus activating it (Park et al., 1999). This is also another cross talking point with ROS-generation pathways that increase oxidative stress in response to toxicants, leading to high production of DAG and subsequent stimulation of melanin production (Costin and Hearing, 2007) (Figure 1). Finally, the cholinergic (muscarinic) and adrenergic (α1) signaling pathways crosstalk with the PKC cascade in response to stimuli (Figure 1) and have been speculated or demonstrated to stimulate the melanin production in skin (discussed later, in Sections 3.8 and 3.9, respectively). Direct impact of tobacco and environmental toxicants on PKC signaling is also lacking, however this is a pathway of much interest to investigate further as it already offers multiple communication points with other routes directly taken by pollutants to act on skin pigmentation.

### 3.4 Wnt/β-catenin signaling pathway; synergy with PKA, Protein Kinase B (PKB) and AhR pathways

The Wnt signaling pathway mediates melanogenesis by enhancing MITF’s expression. As part of this cascade, Frizzled (FZD) receptors bind to the transmembrane molecule Low-Density Lipoprotein Receptor-related Protein (LRP5/6) to form the LRP-FZD dimer complex that modulates cell differentiation and proliferation (Nusse and Clevers, 2017). Wnt’s expression is upregulated by exposure to UV (Yamada et al., 2019) and in response it connects with the LPR-FZD complex. This binding stabilizes the cytoplasmic β-catenin which then translocates to the nucleus where it binds with the T Cell/Lymphoid Enhancing (TCF/LEF) transcription factor to synergistically upregulate MITF gene expression (Takeda et al., 2000; Steingrímsson et al., 2004; Pillaiyar et al., 2017) (Figure 1), with direct downstream upregulating effects on melanogenic enzymes. This pathway cross talks with multiple others to impact melanin production: AhR signaling pathway (Bahraman et al., 2021) (Section 3.1), PKA cascade through α-MSH (Yamaguchi and Hearing, 2009) (Section 3.2), and PKB pathway through the Glycogen Synthase Kinase (GSK)-3β route (detailed herein).

The intricacies of AhR and Wnt/β-catenin pathways crosstalk are still not fully understood. Several studies using animal test systems or liver cells supported the impact of TCDD from tobacco smoke on AhR activation regulated by the Wnt/β-catenin signaling (Mathew et al., 2008; Procházková et al., 2011). Previous publications reported on a direct connection between the components of this intracellular route whereby β-catenin’s expression was strongly induced in melanocytes after exposure to AhR ligands (Nakamura et al., 2013). Another entry of AhR to the Wnt/β-catenin pathway is governed by the α-MSH-induced PKA cascade (Bahraman et al., 2021). These studies providing direct or indirect links between multiple signaling routes exemplify the complexity of the intracellular pathways contributing to melanin production as a response of melanocytes to stress factors.

Another crosstalk is based upon Wnt’s activity on the PKB pathway: when Wnt acts on its cell surface receptor LRP/FZD (He et al., 2004), Dishevelled (Dvl) cytoplasmic phosphoprotein acting directly downstream to FZD induces the accumulation of β-catenin in the cytoplasm by inhibiting the GSK-3β-dependent phosphorylation of β-catenin (Kishida et al., 2001). Accumulated β-catenin is then translocated into the nucleus where it follows the path described above to stimulate MITF and subsequently the melanogenic pathway. Also acting at this crossroads is TCDD, the high-affinity ligand of AhR, which was shown to be able to increase the phosphorylation level of PKB and GSK-3β (Xu et al., 2014) and to activate β-catenin (Al-Dhfyan, 2017). This crosstalk between AhR and PKB/GSK-3β/β-catenin pathway remains not thoroughly understood and likely not fully explored. To complicate the mechanistic profile even further, it has been reported that AhR could activate PKB in a ROS-dependent manner (Liao et al., 2014) or through binding Src kinase in the cell membrane (Ye et al., 2018). All these interactions between factors and intracellular routes have been supported by experiments in various cells lines, with no direct relation to keratinocytes or melanocytes. Therefore, work to verify them in skin lineages should be conducted to determine any direct implications on skin pigmentation in response to pollutants and tobacco. Other mechanisms are also possible and further investigations are surely needed to clarify the complexity of communication between these pathways.

### 3.5 Oxidative stress pathways

Air pollutants become bioavailable to the skin layers *via* nanoparticles and play a key role in the synthesis of quinones which are responsible for the production of ROS. Increased amounts of ROS and free radicals within the cells overcome skin’s innate enzymatic (glutathione peroxidase, glutathione reductase, superoxide dismutase, and catalase) and non-enzymatic antioxidant defense systems (vitamin E, vitamin C, and glutathione) (Thiele et al., 1997). The interaction of ROS and free radicals with the lipid-rich plasma membranes initiates a lipid peroxidation reaction that ultimately triggers an increase of metalloproteinases (MMPs) (Farage et al., 2008), which represent a key cross talking point between multiple pathways. ROS also stimulate the release of pro-inflammatory mediators, which results in a vicious cycle of inflammation and metabolic impairments, with downstream damaging activities on skin pigmentation (cross talking, see Section 3.6). Therefore, ROS are at the crossroads of multiple physiological pathways directly or indirectly impacted by environmental stimuli.

Epidermal melanocytes are especially susceptible to high ROS concentrations due to their specialized function to synthesize melanin through a process that itself induces oxidative stress. ROS are generated in the skin from sources that can be extrinsic (UV, pollution, tobacco) and intrinsic (metabolically generated pro-oxidants), and were shown to accelerate skin pigmentation in experiments using antioxidants. Keratinocytes adjacent to melanocytes have a significant contribution to UV-induced skin pigmentation. Furthermore, H_2_O_2_ (generated by UVB irradiation) (Schallreuter et al., 2004) activates epidermal phenylalanine hydroxylase, which produces L-Tyrosine from L-Phenylalanine, and thus contributes to melanogenesis by increasing the concentration of L-Tyrosine, the initial substrate of TYR (Figure 1—Melanin Synthesis Pathway). Additional studies have also demonstrated another mechanism involving H_2_O_2_ in the regulation of TYR *via* p53 through transcription of the hepatocyte nuclear factor (HNF)-1α, which in turn can also affect the POMC response (Schallreuter et al., 2008), with downstream action on the PKA pathway (see Section 3.2). Many studies reported that UV can induce the formation of ROS and Reactive Nitrogen Species (RNS) that act as signaling messengers to stimulate melanin production (Mastore et al., 2005; Dong et al., 2010; Kim and Lee, 2013) by increasing the amount of TYR and TYRP-1 (Sasaki et al., 2000). While the molecular mechanisms are still subject to investigation, studies showed that nitric oxide (NO) may play a vital role in the activation of TYR through guanylate cyclase and guanosine 3′,5′-cyclic monophosphate (cGMP)-dependent protein kinase (PKG) or by increasing its expression in melanocytes (Sasaki et al., 2000).

Through crosstalk, ROS activate the MAPK family subsequently inducing the activator protein 1 (AP-1), a transcription factor which plays an essential role in the transcriptional regulation of MMP-1, MMP-3, MMP-9, and MMP-12, found to be elevated in aged human skin (Shin et al., 2015; Mujahid et al., 2017). Nuclear factor-κB (NF-κB) is another transcription factor activated by ROS (Cui et al., 2007) and known to mediate the responses to UV irradiation and photo-aging, primarily by upregulation of MMP-1 and MMP-3 in dermal fibroblasts (Pittayapruek et al., 2016). In relation to pigmentation, upon melanocytes activation, their migration is initiated by the decoupling from the basement membrane (Haass and Herlyn, 2005) and further from keratinocytes (Birlea et al., 2017). MMPs induce the decoupling of melanocytes from keratinocytes and coordinate the attachment of melanocytes to the next epidermal unit. Therefore, it is conceivable that pollutants could impact this pathway and ultimately play a role in the associated stimulation of melanin synthesis, however more research in this regard is needed. For simplicity, these possible pathways are not included in Figure 1.

Of the pollutants known to impact the skin, BaP and hazardous dioxins activate AhR with a robust ROS generation, leading to acne and chloracne (Tsuji et al., 2011; Furue et al., 2014). The BaP/AhR signal also induces CYP1A1 (Li et al., 2003; Tsuji et al., 2011; Mescher et al., 2019), which further converts PAHs to quinones capable to upregulate ROS synthesis (Li et al., 2003). Furthermore, PM2.5 contains environmental metals and PAHs (Nel et al., 2006) that may increase the PAH-induced ROS generation (Chen and Lippmann, 2009; Magnani et al., 2016). Further supporting evidence was reported by Murphy et al., 2004 showing that the AhR pathway is activated by both TCDD and all-trans retinoic acid which increase MMP-1 expression in normal human keratinocytes. TCDD-induced expression of MMP-1 through AP-1 route (Murphy et al., 2004). Human melanocytes and some melanoma cell lines are activated by TCDD, and were shown to express both AhR and ARNT, and to increase MMP expression and activity following the exposure to toxicants (Villano et al., 2006). All these interactions are cross talking points between complex pathways that pollutants follow to impact melanin production in the skin.

In terms of tobacco’s participation to skin pigmentation, significantly increased levels of MMP-1 mRNA were observed in the dermal connective tissue of smokers compared to nonsmokers in a clinical study (Lahmann et al., 2001). Tobacco smoke extract induced the production of MMP-1 and MMP-3 and resulted in abnormal regulation of extracellular matrix deposition in human cultured skin fibroblasts (Yin et al., 2003). Moreover, tobacco smoke is a major source of PAHs exposure in humans. In this regard, it has been shown that tobacco smoke extract induced MMP-1 expression *via* activation of AhR signaling pathway in human fibroblasts and keratinocytes (Ono et al., 2013). Supportive evidence regarding the contribution of pollutants and tobacco to skin hyperpigmentation *via* ROS is also provided by multiple other studies (Vierkötter et al., 2010; Cho et al., 2012; Ishiwata et al., 2013; Delijewski et al., 2014; Peng et al., 2017; Leijs et al., 2018; Milani et al., 2019; Khmaladze et al., 2020; Krutmann et al., 2020; Suo et al., 2020; Yang et al., 2020; Chan et al., 2021; Grether-Beck et al., 2021; Moon et al., 2022) discussed in detail in Table 1.

### 3.6 Inflammation pathways

Skin exposure to air pollution toxicants induces mechanisms of cell detoxification which are active over extended periods of time and lead to elevated ROS levels and lipid peroxidation, resulting in skin alterations, including hyperpigmentation (Vierkötter et al., 2010; Patatian et al., 2021). Air pollution also induces inflammation, activates the AhR pathway, and leads to multiple alterations in skin’s physiology (Cho et al., 2012; Mancebo and Wang, 2015; Damevska et al., 2021; Nkengne et al., 2021). It seems that AhR is at the crossroads of these multiple pathways as it was shown to determine the severity of symptoms in chronic skin inflammation (Di Meglio et al., 2014). Its activation is essential for melanocyte survival and melanogenesis, which can be linked to the appearance of senile lentigines (Nakamura et al., 2015).

Recent research has shown that IL-33 stimulates phosphorylation of p38 and CREB, and consequently increases the expression of TYR, TYRP-1 and DCT through MITF, resulting in augmentation of melanogenesis (Zhou et al., 2014). Abundant IL-33 mRNA was found to be induced by UVB in human keratinocytes (Meephansan et al., 2012) and fibroblasts (Arend et al., 2008; Byrne et al., 2011), suggesting that skin’s response to stress factors leads to IL-33 release and ultimately to skin pigmentation. By involving mast cells (Ali et al., 2007; Moulin et al., 2007), macrophages (Schmitz et al., 2005), CD 4+T cells, basophils, dendritic cells and neutrophils, IL-33 is likely promoting Th2-skewed skin inflammation, which is another indirect effect on pigmentation (Hsu et al., 2020). Through activation of NF-kB, IL-33 may be able to regulate outcome of diseases such as atopic dermatitis, which can ultimately trigger pigmentation (Cevikbas and Steinhoff, 2012).

IL-18 is known to modulate both innate and adaptive immunity, and its dysregulation can cause autoimmune or inflammatory disease. Recent studies suggest its participation in regulation of pigmentation based on its activity to promote melanogenesis through upregulation of TYRP-1 and DCT expression (Zhou et al., 2013; Zhou et al., 2016), and by activating pathways that ultimately increase MITF’s expression. Furthermore, Chen et al., 2010 showed that the combination of keratinocyte growth factor (KGF) and IL-1α increased melanin deposition and is responsible for initial stage of human solar lentigines.

PGE2 is released in significant amounts by keratinocytes exposed to UV and in inflammatory conditions such as wound healing, and stimulates the formation of dendrites in melanocytes (Pentland et al., 1990; Pentland and Mahoney, 1990). PGE2 is released by keratinocytes and melanocytes through a process controlled by phospholipase A2 (PLA2), COX and prostaglandin E synthase (Gledhill et al., 2010). PGF2α is produced by fibroblasts and keratinocytes and was shown to stimulate melanocyte dendrite formation and TYR activation (Scott et al., 2005). Therefore, prostaglandins are potent inflammatory mediators with demonstrated activity on the melanogenic pathway and offer an established route for any pollutants to act upon. Further targeted experiments in this regard should be conducted to confirm this hypothesis.

Besides lentigines, post-inflammatory hyperpigmentation (PIH) is another skin condition typified by dark and flattened spots on the body (Markiewicz et al., 2022). Uneven skin tone is caused by exaggerated melanin production, often further aggravated by UV exposure. Inflammatory markers can also trigger PIH associated with skin conditions such as acne, atopic dermatitis, etc. (Kaufman et al., 2018). The PIH profile is characterized by production of cytokines, chemokines, and ROS during the inflammation that ultimately stimulates melanocyte growth, melanin production and its transfer to keratinocytes. Factors promoting these inflammatory events are EGF, IL-1, IL-6, tumor necrosis factor (TNF), leukotrienes (LT)C4 and LTD4, prostaglandins E2 and D2, and thromboxane-2 (Taylor et al., 2009). All these factors intersect on multiple other pathways that directly or indirectly augment the production of melanin in the skin and open wide the gates for environmental stimuli to enhance the pigmentation and accelerate skin aging. The direct connections however still await confirmation through thorough research that can be accomplished by NAMs that cast a wide mechanistic net and provide needed clarifications.

### 3.7 Nicotine: affinity to melanin

A significant body of evidence is reported in the scientific literature highlighting important aspects of the relationship between nicotine and melanin, primarily based on animal studies (Tsujimoto et al., 1975; Leeds and Turner, 1977). Nicotine itself and other tobacco constituents, specifically the carcinogens N′-nitrosonornicotine (NNN), 4-(methylnitrosamino)-1-(3-pyridyl)-1-butanone (NNK), and BaP, have been shown to accumulate in tissues containing melanin (Domellöf et al., 1987). Although many studies demonstrated that tobacco smoke causes skin aging mediated by its effects on fibroblasts and keratinocytes (Morita, 2007; Morita et al., 2009; Ono et al., 2013), narrowing of blood vessels (Argacha et al., 2008) and loss of vitamin C (reviewed by Carr and Rowe, 2020), only recently several studies reported on the mechanisms underlying smoker’s pigmentation.

Nicotine was shown to have affinity for melanin partly due to its precursor role in the pigment synthesis (Mizuno et al., 1997) and to its irreversible binding to the complex melanin molecules (King et al., 2009; Delijewski et al., 2014; Tamai et al., 2014). Nicotine and tobacco-specific toxic byproducts may also be trapped in tissues containing melanin (see also Section 3.10 regarding the possible contribution of adipose tissue), which results in extended exposure and associated health issues noticed in smokers (Szüts et al., 1978; Szparaga et al., 2021). The activity of melanogenesis enzymes may be a factor contributing to the release or degradation of toxic tobacco compounds (Brittebo and Tjälve, 1981). The role of melanin in tissue uptake of nicotine and tobacco-specific toxicants may be of particular concern to individuals with highly pigmented skin (Yerger and Malone, 2006). It was also shown that nicotine has the ability to form adducts with DOPAquinone, a precursor of melanin production (Dehn et al., 2001). Furthermore, nicotine can be incorporated in melanin molecules during hair formation (Stout and Ruth, 1999).

### 3.8 Cholinergic pathway

The cholinergic signaling system (nicotinic and muscarinic receptors) plays an important role in keratinocytes and melanocytes physiology (Kurzen et al., 2007; Wu et al., 2020a; Wu et al., 2020). Nicotine and related byproducts are known to impact the skin primarily by activating nicotinic acetylcholine receptors (nAChRs) (Grando et al., 2006) and downstream by mediating their complex actions in tobacco users (Benowitz and Hukkanen, 2009). Nicotine was shown to induce pigmentation in oral mucosal lesions following application of sublingual tablets (2 mg) for 3–6 months in a smoking cessation study (Wallström et al., 1999). Nicotine was also found to induce melanosome dispersion in dermal melanocytes (melanophores) of a teleostean fish (Visconti et al., 1984).

Within the cholinergic system, α7 nAChR plays a key regulatory role in modulating melanosome uptake in keratinocytes being induced by UV exposure (Figure 1). The exposure to UV is a stimulus to release acetylcholine from keratinocytes (Wu et al., 2020a; Wu et al., 2020), which further triggers the activation of α7 nAChR, localized on the surface of keratinocytes, leading to the intracellular influx of calcium. The mobilization of calcium ultimately initiates phagocytosis of melanosomes by keratinocytes (Guo et al., 2023).

While melanocytes appear to lack α7 nAChR, primary human sebocytes *in vitro* and sebaceous glands were reported to express the receptor (Li et al., 2013), which leaves room for speculation in terms of the contribution of adipose tissue to pigmentation when exposed to tobacco or other pollutants (see Section 3.10). Therefore, the evidence available thus far indicates that the impact of nicotine on pigmentation is mediated primarily by keratinocytes and their response to stimuli. Interesting reports showed some possibilities of signaling mediated by α7 nAChR however provided by cell lineages other than melanocytes. For example, the receptor was shown to transduce signals through the PI3K cascade in rat neuronal cells (Kihara et al., 2001), a path that in melanocytes acts directly onto MITF (see Section 3.4). Liu et al., 2017a showed using dopaminergic neurons that α7 nAChR mechanisms play a key role in a Parkinson’s disease mouse model *via* regulation of the Wnt/β-catenin signaling pathway, known to also impact pigmentation through the PKA and AhR synergistic effects. Since melanocytes derive from neural crests, this association can be explored from the perspective of the nicotinic receptors to determine if tobacco and pollutants can directly join the pathway and impact the melanin production in skin as a result.

### 3.9 Adrenergic pathway

The autocrine adrenergic intra- and intercellular signal transduction network in the human epidermis contributes significantly to the regulation of vital functions within the epidermal melanin unit. The epidermal adrenergic signals control multiple cellular processes such as cell growth and differentiation, motility, calcium homeostasis, and pigmentation *via* the α1-and β2-adrenoceptors.

It was established that human epidermis has the capacity to synthesize catecholamines (norepinephrine and epinephrine) from the essential amino acid L-phenylalanine. The first step leads to the production of L-Tyrosine catalyzed by phenylalanine hydroxylase in the presence of iron, molecular oxygen, and the cofactor/electron donor (6R)-L-erythro-5,6,7,8-tetrahydrobiopterin, followed by the rate-limiting step *via* tyrosine hydroxylase controlled by the same cofactor (Nagatsu et al., 1964) (Figures 1 – Catecholamines Synthesis). The binding of catecholamines to the β2-adrenergic receptor triggers the activation of cAMP production and the downstream PKA signaling pathway (cross talk, see Section 3.2) (Figure 1). Gillbro et al., 2004 demonstrated that a specific functional β2-adrenergic signal exists in human melanocytes leading to pigmentation through the cAMP/PKA pathway, which is now considered the main axis for the catecholamine control of melanogenesis. In a cross talking manner, the epidermal β2-adrenergic signaling also increases cAMP in response to release of epinephrine by keratinocytes, which results in calcium increase *via* activated PKC (Schallreuter et al., 1992; Koizumi et al., 1997). Therefore, cAMP can be considered the liaison between the PKC and PKA pathways.

It was also established that human melanocytes express α1-adrenoceptors, which are induced by norepinephrine yielding the IP_3_ and DAG signals (Figure 1). While the direct link between nicotine and adrenergic receptors in the skin is not supported currently by publications, indirect evidence comes to make the possibility worth investigating it further. For example, Choi et al., 2020 demonstrated the anti-melanogenic potential of carvedilol, a nonselective beta blocker with weak α1-blocking activities. Carvedilol reduced melanin content and cellular TYR activity without impact on cellular viability of human and mouse melanocytes. The compound also downregulated MITF, TYRP-1, DCT, and CREB.

Furthermore, the catecholamine synthesis pathway (Figure 1) uses Dopamine which is also involved directly in the melanin synthesis; therefore, increase in Dopamine in response to stimuli could increase the melanin production directly or indirectly.

### 3.10 Melanogenesis and the contribution of adipose tissue

PCBs and similar derivatives such as dioxins, polybrominated diphenyl ethers (PBDEs), hexabromocyclododecane (HBCD) etc. are often grouped under the umbrella of POPs. Included in this group even though it is not itself a POP is the endocrine disruptor bisphenol A (BPA) which acts as a POP. By sequestering POPs, the adipose tissue can protect other organs and tissues from toxic overload, however this storage may prove in long term detrimental to the body. A relatively recent study (Liu et al., 2017b) showed that PM2.5 inhibited sebocyte proliferation, reduced lipid synthesis and induced inflammatory cytokines IL-1α, IL-6, and IL-8. Additionally, the expression of AhR, ARNT, cytochrome P450 1A1 was significantly increased following PM2.5 exposure.

Even though the impact of pollutants on various cell lines was investigated, experiments targeting the adipose tissue are rather rare. Going further, from a pigmentation perspective, the contribution of adipose tissue is often overlooked and seldom researched with few exceptions. For example, Baranova et al., 2005 reported a statistically significant overexpression of *TYR*, *DCT*, melanosome transport protein RAB27a, and melan-A (MLANA) in visceral adipose tissue of morbidly obese patients. A later follow-up study by Randhawa et al., 2009 confirmed the expression of TYR, DCT, TYRP-1, and MITF and subsequent melanin biosynthesis in adipose tissue. Furthermore, TYR activity in the adipose tissue samples was much higher compared to samples collected from non-obese subjects. One of the limitations of investigating the impact of pollutants on pigmentation in adipose tissues is given by the incapacity of cultured adipocytes to sustain the enzymatic activity of TYR or the production of melanin, nor adequate post-translational modifications or the correct folding of TYR. We consider the participation of the adipose tissue to the melanin synthesis and its significant role in storing POPs with demonstrated impact on melanogenesis an avenue yet to be fully explored (see also Section 3.8 for further speculations on sebocytes possible contribution to pigmentation *via* the cholinergic pathway as they express the α7 nAChR).

### 3.11 IRE1 pathway involvement in correct folding of melanogenic enzymes

Proteins’ accumulation in the endoplasmic reticulum (ER) is a trigger of the unfolded protein response (UPR). Inositol-requiring protein 1 (Ire1), Protein Kinase R (PKR)-like endoplasmic reticulum kinase (Perk), and activating transcription factor (Atf)-6 are components of the UPR (Bertolotti et al., 2000). One very recent study showed that PM can increase melanin production in melanocyte, mouse skin, and human skin models. The expressions of unfolded protein response molecules was also increased in response to PM exposure. The IRE1α signaling pathway was consistently upregulated and it was shown to be further related to PM-triggered melanogenesis (Ahn et al., 2022).

PM treatment of melanocytes induced cAMP and the expressions of phosphorylated Calcium-Calmodulin-dependent Protein Kinase II (CAMKII) and CREB. In parallel, PKA was shown to induce IRE1α phosphorylation (Mao et al., 2011; Asada et al., 2015) which in turn phosphorylates CAMKII and CREB (Kim et al., 2010; Vo et al., 2019). Based on these discoveries, it was hypothesized that PM induced melanogenesis through PKA signaling in a cross talking manner with the IRE1α signaling pathway (Ahn et al., 2022). These preliminary results make the investigation of the folding pathways appealing in the attempt to determine if a direct impact of pollutants and tobacco exists, with downstream effects on skin hyperpigmentation. For simplicity this speculative pathway is not presented in Figure 1.

Investigation of Tyr folding in mouse melanoma cells was conducted using *N*-butyldeoxynojirimycin (*N*B-DNJ), an N-alkylated imino-sugar analog of glucose which inhibits α-glucosidase I and II activity. As a result, most glycans on Tyr molecule are arrested as glycosylated structures and prevented from undergoing any further processing (Branza-Nichita et al., 1999). This inhibition of early N-glycan processing in the ER may impact the maturation of the amino acid chain by preventing the correct association with ER chaperones calnexin/calreticulin responsible for folding. This study showed that *N*B-DNJ dramatically impacts the folding and maturation of Tyr. As a consequence, its enzymatic activity was almost entirely abolished, resulting in a complete loss of pigmentation in the treated cells. However, the enzyme was correctly transported to the melanosomes. It should be pointed out that *N*B-DNJ is the active ingredient in Zavesca (miglustat), which is an oral drug used to inhibit the biosynthesis of macromolecular substrates that accumulate pathologically in glycosphyinolipidoses such as type I Gaucher disease. While repurposing of drugs is a common practice in pharma, allowing an existing licensed drug to be reused for a different indication, this step has never been taken for cosmetic purposes. Since imino-sugar derivatives may hold potential as depigmenting agents and they could be considered as cosmetic candidates for the treatment of various pigmentary conditions such as senile lentigines, melasma, etc. (Costin et al., 2002), this possibility of repurposing is intriguing and appealing.

## 4 Testing platforms used to study pigmentation in the context of skin aging induced by pollution and tobacco

Manufacturers of products designed to address the damaging effects of pollutants and tobacco on skin face increasing demands for safe and efficacious products in response to population’s awareness of poor environmental conditions and their impact on life quality. Designing products that target modulation of skin tone is particularly challenging given the wide range of ethnic skin types. This is an aspect that needs to be taken into account in addition to variation in seasonal weather patterns, differences in life styles and personal care and cosmetics usage and associated benefit expectations (Mistry, 2017). Society nowadays exercises a sophisticated inquisitiveness exemplified by demands for more ethical, natural formulations in cosmetic research, supported by mechanistic rationales to motivate the consumer consider the next product on the market (Juliano and Magrini, 2017). While challenging to explain complex molecular pathways in marketing terms accessible to masses, this demand is to the advantage of the scientists who reported multiple lines of evidence not only regarding the mechanisms of pollutants’ action onto skin and pigmentation, but also explored those mechanisms to design potent ingredients and finished products. Leveraging the existing population habits and an in-depth understanding of cellular and molecular mechanisms that contribute to the efficacy of products helped science deliver anti-pollution benefits.

Currently, there are no established standardized anti-pollution tests or international guidelines for product development. The many different types of methodologies we discussed thus far (*in vitro*, *ex vivo*, *in vivo* and clinical) and summarized in Table 1 are usually designed as fit for purpose and may be used to support efficacy claims addressing tobacco use or pollution exposure as causes of hyperpigmentation which is a hallmark of skin aging. Many brands rely on supplier data (Spencer, 2016; Whitehouse, 2016) while others use post-marketing surveillance data for claims support. However, *in vitro* and clinical test data should be used as they provide direct support to establish safety and efficacy of products with biological activity. As such, we will attempt in this section of the manuscript to narrow down the contender methods and endpoints that could be used as a portfolio of tests to research the impact of pollution and tobacco on skin and to support the efficacy of actives to counteract their damaging effect on pigmentation.

### 4.1 Position of NAMs in the cosmetic science research framework

Our analysis of the studies reporting data regarding the impact of pollution and tobacco on skin pigmentation revealed two key aspects: 1) there is more research conducted and/or published on pollution compared to tobacco, with a visible focus on PM; and 2) overall, the NAMs are used more frequently compared to animal or clinical studies, which is an approach we encourage to continue and be further explored.

For example, we identified 16 manuscripts reporting on pollution’s contribution to skin hyperpigmentation of which 11 addressed the PM (and 2 of those were specifically on DEPs), 4 were on PAHs and 1 for PCBs (Table 1) in terms of the type of toxicant. Regarding the study design, the manuscripts reported a total number of 32 experiments conducted as follows (determined using the length of treatment column): 7 clinical studies, 15 cell-based methods, 2 based on reconstructed tissue models, 4 on *ex vivo* explants and 4 using animal test systems. As already mentioned, PM was thoroughly investigated using a comprehensive portfolio of *in vitro*, *in vivo*, *ex vivo* and clinical approaches, thus providing a fuller picture on how PM impacts skin pigmentation, at a macroscopic and molecular, mechanistic level. This approach based primarily on NAMs should be expanded and applied for the research of other pollutants in order to obtain fast, human-relevant, reliable mechanistic insights. The cell-based assays used to investigate pollution’s contribution to hyperpigmentation were based on primary or immortalized human keratinocytes or melanocytes of various degrees of pigment producing capacity; in rare cases mouse melanocytes were used (Krutmann et al., 2008; Luecke et al., 2010; Abbas et al., 2017). In general, the endpoints of interest were conducted for safety assessment (viability, apoptosis) or to gain mechanistic knowledge on pollution’s impact on the key players of the melanogenic pathway. Of these assays, many were biochemical (enzymatic activity, melanin content) (Suo et al., 2020; Shi et al., 2021), histological, based on gene and protein expression (Suo et al., 2020; Yang et al., 2020; Shi et al., 2021; Yang et al., 2022), including key markers of various intracellular pathways. Of similar versatility in terms of endpoints and data output were the *ex vivo* platforms and those based on reconstructed tissue models (Krutmann et al., 2020; Grether-Beck et al., 2021). The animal studies usually employed histological endpoints or assessed melanin content, melanocyte density (Krutmann et al., 2008; Abbas et al., 2017), while clinical studies were focused primarily on the impact of pollutants on skin color at a macroscopic level (Vierkötter et al., 2010; Peng et al., 2017; Nkengne et al., 2021) (refer to Section 4.3; Subsection 4.3.3. Endpoints for more details on endpoints).

For tobacco research analyzed in our manuscript (Table 1), the majority of studies were clinical (8), with a small number of pre-clinical studies (3) based on cell culture models using human melanocytes to address the effect of cigarette smoke (Nakamura et al., 2013) and nicotine (Delijewski et al., 2014) on enzymatic activity relating to melanin production. While the majority of the clinical studies address skin pigmentation on a surface level, few others have collected more than “skin deep” measurements by analyzing bodily fluids for enzymes (King et al., 2009; Dalrymple et al., 2022) and metabolites (Liakoni et al., 2019).

Our analysis draws encouraging conclusions regarding the importance and position of NAMs in cosmetic research focused on environmental toxicants and provides an up-to-date view to support the advances made by this field relying less and less on animal testing. Using NAMs provides several advantages over animal or clinical studies to evaluate the effects of air pollutants and tobacco on skin pigmentation and aging. Multiple alternative methods based on the 3Rs were developed in response to increasing ethical concerns regarding animal experimentation (Russell and Burch, 1959). The 3Rs principles were first used in 1998 when the United Kingdom implemented the first total ban on animal-tested cosmetic products. Besides overcoming ethical concerns related to animal test systems, *in vitro* methodologies offer other scientific advantages over animal studies such as reproducibility and high throughput, which support complex mechanistic studies to investigate the activity of toxicants on skin and the design of actives to counteract them. Animal or clinical studies are reliant on availability of animal species and human volunteers, which can increase the time to recruit and conduct these lengthy studies that provide limited molecular insights about cellular pathways. Animal testing can be performed on a variety of species, however the results are not entirely reflective of a human skin exposure mainly because of the differences in architectures and immune responses between animal and human skin. With careful selection, proper training and skillful handling, NAMs are now indispensable for modern dermatology and skin aging research. *In vitro* anti-pollution tests facilitate a deeper understanding of changes occurring in the skin cells. One of the biggest advantages of these assays is the control of specified conditions and the endless possibilities to customize the testing parameters to fit the purpose of the experiments. In an animal test system or in clinical studies, this flexibility is not always possible, thus limiting the mechanistic data output.

### 4.2 Use of NAMs to design potent actives counteracting effects of pollution and tobacco on skin pigmentation

We integrated in Table 1 the existing studies that took advantage of the knowledge reported on mechanisms of action used by pollution and tobacco on skin pigmentation to design efficacious actives to counteract their deleterious effects. We identified a total of 5 reports and all of those only addressed the efficacy of actives on pollution exposure and impact on skin pigmentation. Of the total of 14 experiments these studies reported on, 1 was a clinical study, 9 were cell-based and 4 used *ex vivo* human explants. Even though based on a relatively small number of studies, our analysis draw several important conclusions as basis for future directions discussed in more details later: 1) efficacy of raw ingredients and finished products to counteract tobacco’s impact on skin pigmentation is either not researched or published on; 2) it is encouraging to see that no animal studies and a limited number (1) of clinical studies were conducted while the vast majority of the results were generated using cell- and explant-based experiments. The limited number of publications is somewhat understood or justified given the competitive market and the need to maintain a reasonable margin without disclosing confidential intellectual property information. However, to the benefit of the consumer, of the science supporting safe and efficacious products, encouraging the scientists to publish will avoid duplicating efforts and delaying launching of important products on the market.

There exist several active compounds, natural extracts and vitamins that have been investigated and showed capacity to inhibit or reverse the effects of pollution on skin pigmentation. Cultural preference plays a major role in the design and priority testing of anti-pollution products. For example, while tanned skin is often considered attractive in the United States, many Asian countries consider fair skin to be preferable (Khmaladze et al., 2020) and hyperpigmentation due to pollution is a concern. Highly populated cities in China, India and Pakistan have a correspondingly high level of pollution which is a growing consumer concern when considering cosmetic products (Mistry, 2017). This growing global awareness of pollution affecting the skin generated an increased cultural demand for anti-pollution products.

As outlined in Table 1, *in vitro*, *ex vivo* and clinical studies have all been used to assess the efficacy of these products. Clinical studies can address changes in skin color and epidermal morphological changes. *In vitro* methods (cell-based) can address pigmentation changes the human eye cannot identify such as gene expression, cell viability or death, cytokine activity and melanin content. *Ex vivo* models can also address many of these “below the surface” activities but can be more difficult to obtain as a reliable test system, in addition to the population variability that should be considered. In this context, science advanced tremendously and provides nowadays commercially available pigmented reconstructed tissue models that can not only eliminate the sourcing issue and population variability, but also offer the possibility to compare models of various phenotypes and explore their responses to toxicants in terms of skin pigmentation (see Section 4.3; Subsection 4.3.1. Test system).

Natural extracts from plants such as *Deschampsia Antarctica* (Milani et al., 2019; Fernández-Martos et al., 2021) and Korean Red Ginseng (Moon et al., 2022) were evaluated for anti-pollution claims. In a clinical study, *Deschampsi Antarctica* reduced dark spots (Milani et al., 2019); the study by Fernández-Martos et al., 2021 supported mechanistically the claims on this natural extract and identified the AhR and inflammatory pathways as those on which the extract is effective. Furthermore, the same study used an *ex vivo* human skin model that was pre-treated with *Deschampsia Antarctica* and then exposed to different pollutants. The pre-treatment prevented downregulation of E-cadherin receptors and overexpression of AhR which were shown to be in response to pollutants (Fernández-Martos et al., 2021). Pre-treatment of human keratinocytes, co-culture of human keratinocytes and melanocytes and murine macrophages with Korean Red Ginseng mitigated the increase of melanin in response to PM10 treatment (Moon et al., 2022). These datasets indicate another key aspect of NAMs: they allow the design of the experiments accommodate a preventive (pre-treatment) or therapeutic (post-exposure treatment) approach which may be difficult to execute in a clinical or animal setting. In this way, the potential of these ingredients to prevent or to treat the effects of pollutants can be addressed and is supported with mechanistic justification.

Besides botanical extracts which are very appealing given their natural origin (for a comprehensive review see Mistry, 2017), various chemically derived actives were of interest and tested using cell-based assays or as formulations using explants. For example, actives A, B, C and D (see Table 1 for definitions) were tested on human keratinocytes, human dermal fibroblasts, human melanocytes and a co-culture of melanocytes and keratinocytes (Khmaladze et al., 2020). In general, a pre-treatment or treatment with the actives upregulated genes involved in the regulation of melanogenic pathway, and inhibited melanin and cytokine expression. A formulation of the active ingredients applied to an *ex vivo* human skin model also inhibited genes related to expression of pigmentation. Vitamins C and E formulated with active ingredients (see Table 1) applied onto an *ex vivo* human skin model reduced hyperpigmentation and gene expression of *MMP-1* (Krutmann et al., 2020).

Overall, the products currently offered on the market address several mechanisms of action in terms of routes by which pollution impacts skin pigmentation: oxidative stress, inflammation, direct action on the melanin pathway (through MITF, Moon et al., 2022), and the AhR governed pathway (Table 1). The fact that the knowledge gained din academic research is now applied to design efficacious products is a huge step, which should be further encouraged, supported and published.

### 4.3 Where do we go from here? Study design: points to consider

Based on our analysis of the pollutants reported to impact skin pigmentation, on the current status of test systems, endpoints and mechanisms of action, we summarize below several key findings and points in support of future directions research could consider in this field.

#### 4.3.1 Test system

When considering the appropriate and relevant experimental design to investigate the impact pollutants and tobacco have on skin pigmentation or to test the efficacy of products with anti-pollution claims, several options exist depending on the endpoint desired. Human immortalized melanocytes and keratinocytes are relatively easy to obtain through established commercial vendors such as American Type Culture Collection (ATCC) (Manassas, VA, United States) and can be maintained for years at a low cost. Specific lines can also be obtained from academic sources, however they need to be carefully and thoroughly characterized and tested for reliability over time. Using a 2D (cellular) model to test anti-pollution properties can provide information on melanin content, cell viability and changes in gene expression. This is a great model when testing individual ingredients for mechanistic purposes as often cellular assays are dilution based to ensure bioavailability of the ingredient to the cells.

Formulations often contain a multitude of ingredients and therefore solubility can be challenging, so a more robust test system such as *ex vivo* or reconstructed (3D) tissue models would be more appropriate. The formulation can be applied directly to the surface of the skin model for a longer duration which would be more reflective of the application of the final product. Using a 3D model can give insight on full penetration of cell layers, anti-inflammatory response, gene expression and cell viability, however they are often more expensive than a 2D model. *Ex vivo* models can provide similar endpoints as a reconstructed tissue model but are limited on donor availability. Clinical studies can also use a final formulation product for multiple applications or longer exposures, however they have several limitations such as expense, variability between subjects, and high variety of protocols used.

There exist several reconstructed human pigmented tissue model systems that could accommodate the testing of cosmetic ingredients and formulations. Suppliers such as BioCell Biotechnology (Guangdong, China - MelaKutis model), Creative Bioarray (Frankfurt am Main, Germany - 3D human Pigmented Skin Model), EpiSkin (Lyon, France - Reconstructed Human Pigmented Epidermis, RHPE, models), MatTek Corporation (Ashland, MA, United States-Melanoderm™), Phenion (Henkel) (Düsseldorf, Germany-epics-M), StratiCELL (Les Isnes, Belgium-various models mimicking pigmentary skin conditions), and TegoScience (Seoul, Korea-NeoDerm-ME) offer these tissue models commercially (Gendreau et al., 2013). Using an established model from a reputable source (*i.e.*, not “home grown”) not only ensures reproducibility in testing but also standardizes the model across multiple laboratories.

At the time of this review, there were no research reports assessing the effect of tobacco on pigmentation using a reconstructed human tissue model. We identify this to be a major area to focus testing of tobacco products on. In addition to the advantages of using a NAM to assess pigmentation changes resulting from tobacco, technology exists that can deliver a full dose of tobacco smoke directly to the reconstructed human tissue model. Smoke chambers such as the Vitrocell Cloud™ chamber (Vitrocell System GmbH, Germany) can be utilized in combination with a 3D model for a robust testing system. Commercially available cigarettes can be “puffed” directly onto the surface of the tissue, replicating a real life exposure to cigarette smoke (Lecas et al., 2016; Prieux et al., 2020; Seurat et al., 2021). After exposure, a variety of endpoints can be used to assess pigmentation changes such as viability, melanin content and distribution, histological analysis and gene expression. This approach has the obvious advantage of replicating *in vitro* the end-user exposure to whole smoke (particulate and gas phases) that other NAMs cannot accommodate.

The NAMs based on 2D and 3D test systems could be used in a tiered approach. Once the individual toxicants/ingredients are assessed in a screening approach using cell-based assays, the final formulation can be applied directly to *ex vivo* or 3D tissues for safety and efficacy investigations using a plethora of endpoints that exist (biochemical, histological, gene expression, etc.). Multiple forms and concentrations of the toxicants/ingredients can be applied topically to the more robust test system, to mimic the end-user exposure, or in the media, to mimic a systemic exposure. These approaches cannot be accommodated in cell-based assays and they represent a great advantage of explants and reconstructed tissue models. A repetitive dose of a cosmetic product can also be applied in a 3D model, aligning more with a real life application and avoiding any challenges of losing the cell population during manipulations, especially with non-adherent cells. After treatment, the media can be collected for subsequent analysis of multiple makers specific to molecular pathways of interest.

Depending on the results of the *in vitro* testing, clinical studies may be applicable. However as described above for the length of time required and reliance on volunteers, clinical testing can be inappropriate if a quick, screening result is desired. Compared to the suite of NAMs we covered in this review, the clinical studies offer the advantage of being able to accommodate a well-rounded testing approach by assessing all parts of atmospheric pollution such as the PM, PAH’s, DEP’s, heavy metals, etc. A clinical study comparing individuals who live in a highly polluted area to those who live in a more rural, less polluted area could provide a fuller picture on the effects of anti-pollution products on the skin (Vierkötter et al., 2010; Peng et al., 2017; Flament et al., 2018; Nkengne et al., 2021). Similarly, tobacco smoke contains more chemical classes than just nicotine and clinical studies can investigate the effects of both the particulate and gas phases. During our analysis, we identified results obtained in seemingly similarly designed clinical studies that were discordant, all of them on tobacco research. For example, the results obtained by Cho et al., 2012 correlated with those obtained by Serri et al., 2010 showing that melanin index values decreased after smoking cessation, however they were not confirmed by Ishiwata et al., 2013 or by Yazdanparast et al., 2019, though the latter investigated the melanin content in the skin of smokers vs. non-smokers (Table 1). Furthermore, studies by King et al., 2009; Tamai et al., 2014 reported correlative results in increased melanin in smokers vs. non-smokers, while the study by Liakoni et al., 2019 did not, likely because in this study the nicotine was administered intravenously.

The clinical studies and NAMs should be used in a tiered, step-wise approach and to fit the purpose of the research. Furthermore, if the possibility of paired *in vitro*-clinical data exists, it should be taken into consideration especially for screening active candidates designed to counteract the impacts of pollutants and tobacco on skin pigmentation. If any NAMs can be identified as good predictors of clinical studies outcome, the strategy can be used to inform clinical studies and reduce their size, scope, price and length.

#### 4.3.2 Treatment options: toxicants, ingredients, and finished products

Both air pollutants and tobacco are characterized by a huge complexity of components which are in a combined particulate and gaseous form. Studies indicated that the gas phase contains the most harmful toxicants produced by combustion. Therefore, to mimic a “real-life” exposure, both phases should be considered as part of hazard and risk assessment. While many studies investigated the mechanisms responsible for occurrence of melasma, lentigines, etc., their conclusions are tightly linked to the form of toxicant used, which in most of the cases overlooked the gas phase. Therefore, this is an area of critical consideration for future research.

In terms of PM in particular, the most common particles used in *vitro* and *in vivo* studies investigating their effects on skin are Standard Reference Materials^®^ from the National Institute of Standards and Technology, 2023 (NIST) (NIST, NP). They represent diesel PM, PM2.5, and a variety of other standards that are geographically specific by collection site. The Joint Research Centre, 2023 (JRC) of the European Commission produces European Reference Materials (ERM) that are used in studies simulating airborne PM (JRC, NP). JRC also provides urban dust particles resembling PM10-like urban dust (with or without PAHs). The National Institute for Environmental Studies (NIES) in Japan offers a PM10-like urban dust certified reference material that was collected from a central ventilating system in a building in Beijing, China (Mori et al., 2008). Several studies reported collection of PM2.5 from seasonal dust storms in Asia and West Africa using an in-house filtering system. Other authors used concentrated ambient air particles (PM2.5) for skin model exposure (Dijkhoff et al., 2020).

In terms of raw ingredients and finished products, industry exercises due diligence in selecting safe and efficacious candidates that are tested for confirmation. Ingredients acting on multiple pathways can be combined and tested for synergistic effects within a finished formula. The ingredients should be tested for toxicity in a model which addresses the activation of these possible pathways to generate a dose response curve, ensuring the ingredient itself is not causing any adverse cytotoxic effects. Once non-toxic concentrations are established, the cells can be pre-treated with the ingredient. The ingredient can be rinsed off and environmental toxins added and compared to a group without pre-treatment for various endpoints such as melanin content and expression of genes encoding for factors relevant to multiple intracellular pathways.

#### 4.3.3 Endpoints

As listed and discussed in detail in Table 1, the endpoints relevant to the pathways governing the melanin production in human skin are of a wide variety. As we indicated in regards to the test system and toxicants thus far, the key is to work with established systems, from commercial sources, with characterized, stable and reliable materials. Given the wide diversity of the endpoints that exist, standardization is lacking in terms of which endpoints to use when action of toxicants or actives on pigmentation is investigated. Industry could take the lead and work together to identify the most relevant and reliable safety and efficacy endpoints. Even if a set of standardized assays would be generated, specific/unique endpoints may still be needed when researching novel pathway that may be of interest. The assays should be qualified for these specific endpoints and cut-off values and ranges established if companies decide using historical databases to deem prototypes safe and efficacious. It is also very important for industry to qualify benchmark materials that can be used to compare the prototypes to in terms of safety and efficacy. As with any assays, proper positive and negative controls should be included in order to assess the performance of each test system and endpoint selected.

Outside the scope of pigmentation itself which was our main interest in this manuscript, other skin aging endpoints can provide insight into how pollution and tobacco affect the skin as a whole. For example, clinical studies often look at multiple skin health factors using the Score of Intrinsic and Extrinsic Skin Aging (SCINEXA) scoring system. This includes measurements of pigment spots but also measures coarse wrinkles, elastosis, telangiectasia, skin laxity and seborrheic keratosis (Vierkötter et al., 2010). Other measurements such as Trans Epidermal Water Loss (TEWL), pH, erythema content, sebum, and skin elasticity and friction can also be performed (Yazdanparast et al., 2019). These are useful for both pollution and tobacco effects because constitutive and facultative skin pigmentation can be measured on one individual but also address population diversity if that is a goal of the research.

The NAMs offer multiple options in terms of endpoints related to skin aging besides those targeting pigmentation. For example, PM exposed to normal human epidermal keratinocytes (NHEKs) decreased the number and size of primary cilia, an organelle responsible for cell to cell signaling for growth factors, nutrients and hormones in the skin (Bae et al., 2019). *Ex vivo* models have investigated how tobacco decreases skin hydrophobicity and induces changes to the lipid bilayer in the *stratum corneum* (Percoco et al., 2021). Hammer et al. (2011) used an *ex vivo* test system (fresh adult full-thickness skin from breast plastic surgery) to investigate the toxicity of cigarette smoke attached to textiles; the study showed that the contaminated textiles represent a potential source of nicotine uptake by skin and induce further adverse effects. Tobacco smoke exposed to a reconstructed human tissue model affected the skin layers, increased production of ROS, and induced an anti-inflammatory response (Lecas et al., 2016). A full thickness human skin model exposed to cigarette smoke (Rasmussen et al., 2010) has demonstrated an increase in ROS generation. Glycation as a reflection of chronological aging was investigated in a reconstructed skin model integrating monocytes in order to address multiple pathways (Pageon et al., 2017). Active compounds (Dead Sea minerals and PolluStop^®^) were evaluated on a reconstructed human tissue model for irritation and inflammation of the skin and found to reduce both when used together (Portugal-Cohen et al., 2017). Feverfew (a natural antioxidant extract) applied to a human skin model (StrataTest^®^) reduced ROS levels elevated by PM exposure (Rasmussen et al., 2010).

Pigmented reconstructed models have been used for endpoints related to skin aging though not directly linked to hyperpigmentation induced by toxicants. Some pigmented 3D models were used to investigate the continuous information exchange within the epidermal melanin unit (Bessou-Touya et al., 1998; Hall et al., 2022). Others became more stable in culture (Gibbs et al., 2000 – for 6 weeks; Zoio et al., 2021-50 days) or more complex through addition of fibroblasts as another conversation partner for keratinocytes and melanocytes and important regulator of pigmentation (Hedley et al., 2002; Zöller et al., 2019). Studies reported on the use of 3D models containing these 3 cell lineages to investigate in-depth the cell-cell, cell-matrix and mesenchymal-epithelial interactions that control skin pigmentation (Duval et al., 2012). Diekmann et al. (2016) took this concept a step further by inducing aging to a model generated with melanocytes, keratinocytes and fibroblasts by using Mitomycin-C which was shown previously to induce accelerated senescence in human dermal fibroblast cultures. Gledhill et al., 2015 investigated the melanin transfer in the first human 3D model generated from induced pluripotent stem cells (iPSCs)-derived fibroblasts, keratinocytes and melanocytes. The advantages of this model are enormous in that the contribution of all these cell lineages is possible and it replicates the native skin environment. The complexity of these models allows investigations into endpoints specific for skin aging. The presence of melanocytes in these test systems should qualify them for experiments investigating the impact of pollutants on pigmentation or to screen ingredients with potential to reverse the activity of toxicants on skin pigmentation.

## 5 Concluding remarks

In this review we provided an overview of the air pollution and tobacco smoke as stress factors that increase pigmentation in the context of extrinsic-induced skin aging and the mechanisms by which they do so. Understanding these mechanisms is of great interest to industries whose main focus is on anti-pollution and anti-tobacco-induced aging skin care and cosmetic products that became an ever increasing necessity due to worsening air quality around the world. In certain cultures, skin color is often related to hierarchies of race, gender, caste and ethnicity and is unavoidably the first phenotypic feature one observes during human interactions given its external positioning within our body. Any minute manipulation of the skin color becomes immediately visible macroscopically since the melanin biosynthesis pathway acts as a barometer in response to voluntary treatments or uncontrollable stress factors. Research into skin pigmentation targets the melanocytes and the tight relationship with the keratinocytes within the epidermal melanin unit. The melanocytes are located deeply in the skin, yet their activity is so highly visible as portrayed by the uniqueness and limitless variety of ethnic skin tones. Equally “praised” for generating beautiful skin color tones and “blamed” for racial discrimination, the inconspicuous melanocytes are probably the most impactful cell type in the human body at a global level. In this manuscript we first traveled deep into the skin to rediscover the melanocytes and identify their response to environmental toxicants. We brought back to surface their message that helps us decipher new mechanisms of actions and molecular pathways and the secrets of how to create solutions in the form of efficacious products. The melanocytes generously equip the scientists with the keys to open the doors to the next-generation of skin lightening actives, the miracle anti-aging cream or natural/non-UV tanning procedure. The possibilities to research a wide variety of pollutants open up an entire new field that awaits for its mysteries be discovered by making use of an equally diverse portfolio of NAMs at our disposal.
